# Supplementary figures and images for: Do more stress and lower family economic status increase vulnerability to suicidal ideation? Evidence of a U-shaped relationship in a large cross-sectional sample of South Korean adolescents
Source: PLoS One. 2021 Apr 26;16(4):e0250794. doi: 10.1371/journal.pone.0250794 (PMC8075251; doi:10.1371/journal.pone.0250794)

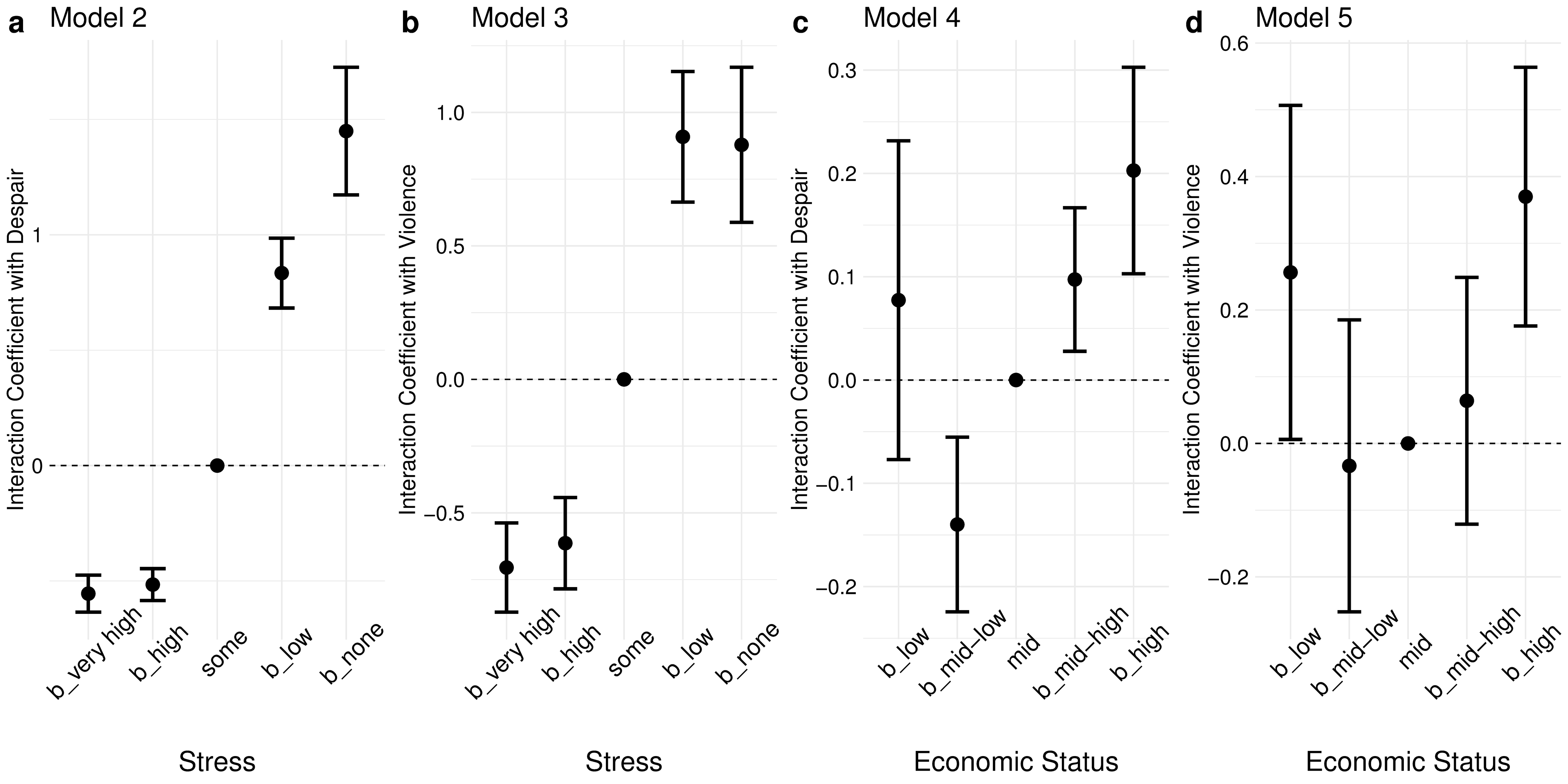

Supplement: S1 Fig — (TIF) [file pone.0250794.s001.tif]
