# Supplementary material for: Do more stress and lower family economic status increase vulnerability to suicidal ideation? Evidence of a U-shaped relationship in a large cross-sectional sample of South Korean adolescents
Source: PLoS One. 2021 Apr 26;16(4):e0250794. doi: 10.1371/journal.pone.0250794 (PMC8075251; doi:10.1371/journal.pone.0250794)
Supplement: S1 Code — (HTML) [file pone.0250794.s002.html]

R-presentation-6.utf8


### Replication R Code for the research article “Do more stress and lower family economic status increase vulnerability to suicidal ideation? Evidence of a U-shaped relationship in a large cross-sectional sample of South Korean adolescents” by Tay Jeong

#### 2021-03-20

```
#Dependencies
library(survey)
library(brms)
library(ggpubr)
library(tidyverse)
library(lattice)
library(stargazer)
library(psych)
library(broom)
library(sas7bdat)
library(data.table)
library(polycor)
library(car)
library(msm)
```

### The dataset

Download the KYRBS rawdata from http://www.kdca.go.kr/yhs/. Download is immediately available for anyone after entering some basic personal details. I downloaded the files in .sas7bdat format and imported them to R. The dataset for each year contains two files, kyrbs201\* and pop1\*. The former is the main dataset with columns indicating survey items and rows individuals. The latter contains the number of schools in each of the 110 strata to be used for finite population correction.

```
#Combine the the two datasets for each year
dat2019 <- merge(kyrbs2019, pop19)
colnames(dat2019)[which(names(dat2019) == "_total_")] <- "_TOTAL_"
dat2018 <- merge(kyrbs2018, pop18)
dat2017 <- merge(kyrbs2017, pop17)

#Assign a different school name to all schools.
dat2018$CLUSTER <- dat2018$CLUSTER + 800
dat2019$CLUSTER <- dat2019$CLUSTER + 800 + 800 #Each year, schools are numbered 1-800. I change that so that there is no overlap.

dat2019$SCHOOL <- paste(dat2019$YEAR, "-", dat2019$CLUSTER) #append the year just to make it extra clear.
dat2018$SCHOOL <- paste(dat2018$YEAR, "-", dat2018$CLUSTER)
dat2017$SCHOOL <- paste(dat2017$YEAR, "-", dat2017$CLUSTER)

#merge the three years' datasets vertically
bigdat <- full_join(dat2019, dat2018)
bigdat <- full_join(bigdat, dat2017)

#copy bigdat to bigdat2s for further processing
bigdat2s <- bigdat

#Suicidal Ideation (Dependent Variable)
bigdat2s$SUI_CON <- car::recode(bigdat$M_SUI_CON, '1=0;2=1') 

#Stress
bigdat2s$STRESS <- car::recode(bigdat$M_STR, ' 5="none" ; 4="low" ;3="some" ;  2="high" ; 1="very high"  ')
bigdat2s$STRESS <- factor(bigdat2s$STRESS, levels=c("some", "none", "low", "high", "very high")) #"some" is the reference
bigdat2s$STRESS_cont <- car::recode(bigdat$M_STR, '5= 0; 4= 1; 3= 2; 2= 3; 1= 4')

bigdat2s$STRESS5 <- ifelse(bigdat2s$STRESS=="very high",1,0)
bigdat2s$STRESS4 <- ifelse(bigdat2s$STRESS=="high",1,0)
bigdat2s$STRESS3 <- ifelse(bigdat2s$STRESS=="some",1,0)
bigdat2s$STRESS2 <- ifelse(bigdat2s$STRESS=="low",1,0)
bigdat2s$STRESS1 <- ifelse(bigdat2s$STRESS=="none",1,0)

#Perceived family economic status
bigdat2s$ES <- car::recode(bigdat$E_SES, '1="high"; 2="mid-high"; 3="mid"; 4="mid-low"; 5="low"')
bigdat2s$ES <- factor(bigdat2s$ES, levels=c("mid", "low", "mid-low", "mid-high", "high"))

bigdat2s$ES5 <- ifelse(bigdat2s$ES=="high",1,0)
bigdat2s$ES4 <- ifelse(bigdat2s$ES=="mid-high",1,0)
bigdat2s$ES3 <- ifelse(bigdat2s$ES=="mid",1,0)
bigdat2s$ES2 <- ifelse(bigdat2s$ES=="mid-low",1,0)
bigdat2s$ES1 <- ifelse(bigdat2s$ES=="low",1,0)

#Recent grief or despair
bigdat2s$SAD <- car::recode(bigdat$M_SAD, '1=0;2=1')
bigdat2s$SAD <- as.factor(bigdat2s$SAD)
bigdat2s$SAD_numeric <- car::recode(bigdat$M_SAD, '1=0;2=1')

#Recent medical treatment due to violence
bigdat2s$VIOLENCE <- ifelse(bigdat$V_TRT==1, 0, 1)
bigdat2s$VIOLENCE <- as.factor(bigdat2s$VIOLENCE)
bigdat2s$VIOLENCE_numeric <- ifelse(bigdat$V_TRT==1, 0, 1)

#Perceived health
bigdat2s$HEALTH <- car::recode(bigdat$PR_HT, '5= "very bad"; 4="bad"; 3="normal"; 2="good"; 1="very good"')
bigdat2s$HEALTH <- factor(bigdat2s$HEALTH, levels=c("normal", "very bad", "bad", "good", "very good")) #"normal" is the reference
bigdat2s$HEALTH_cont <- car::recode(bigdat$PR_HT, '5= 0; 4= 1; 3= 2; 2= 3; 1= 4')
bigdat2s$HEALTH_cont <- ( bigdat2s$HEALTH_cont - mean(bigdat2s$HEALTH_cont) ) / sd(bigdat2s$HEALTH_cont) #standardize

#Female
bigdat2s$SEX <- car::recode(bigdat$SEX, '1=0; 2=1')

#GRADE
bigdat2s$GRADE_cont <- bigdat2s$GRADE - mean(bigdat2s$GRADE) #original variable is from 1 to 6. Treat it as continuous and center it at zero.

#Residential type
bigdat2s$RESIDENTIAL <- car::recode(bigdat2s$E_RES, '1="family"; 2= "relative" ; 3="dorm/studio"; 4="dorm/studio"; 5 = "nursury/orphange"')
bigdat2s$RESIDENTIAL <- factor( bigdat2s$RESIDENTIAL, levels = c("family","relative","dorm/studio","nursury/orphange")  ) #family is the ref cat.

#divide weights by 3 as three years' data have been merged (according to KYRBS 2019 user's manual p.58)
bigdat2s$W <- (bigdat2s$W)/3
```

```
head(bigdat2s[, c("SCHOOL", "SUI_CON", "VIOLENCE", "SAD", "STRESS", "ES","HEALTH_cont", "GRADE_cont", "SEX", "RESIDENTIAL", "STRATA", "W")])
```

```
##        SCHOOL SUI_CON VIOLENCE SAD STRESS       ES HEALTH_cont GRADE_cont SEX RESIDENTIAL STRATA        W
## 1 2019 - 1711       0        0   0   some      mid -2.17060180  0.4967014   0      family 2019_1 29.09786
## 2 2019 - 1711       0        0   0   some      mid -1.04071406  0.4967014   0      family 2019_1 29.09786
## 3 2019 - 1711       0        0   0   some      mid  1.21906143  0.4967014   0      family 2019_1 29.09786
## 4 2019 - 1711       0        0   0   some  mid-low -1.04071406  0.4967014   0      family 2019_1 29.09786
## 5 2019 - 1711       1        0   1   high mid-high  1.21906143  0.4967014   0      family 2019_1 29.09786
## 6 2019 - 1711       0        0   0   some mid-high  0.08917369  0.4967014   0      family 2019_1 29.09786
```

### Descriptive Statistics

Describe the distribution of key variables (Fig 1). Font sizes used for plots inserted in the article were changed to fit the html presentation.

```
#Suicidal Ideation
bigdat2s$SUI_CON.factor <- factor(bigdat2s$SUI_CON, levels=c("0", "1"))
p.SI <- bigdat2s %>%
  count(SUI_CON.factor) %>%
  mutate(prop = n/sum(n)) %>%
  ggplot(aes(x=SUI_CON.factor, y=n)) + 
  labs(x = "Suicidal Ideation", y = "Number of Observations") +
  theme(legend.position = "none") +
  theme(axis.text.x = element_text(size=10, color = "black"), axis.text.y = element_text(size=10, color = "black")) +
  theme(axis.title.x = element_text(size=12, color = "black"), axis.title.y = element_text(size=10, color = "black")) +
  geom_col(aes(fill = SUI_CON.factor), position = "dodge",width=0.5) +
  geom_text( aes(label = scales::percent(prop), y = n), 
             size = 3,
             position = position_dodge(width = 0.9),
             vjust = 1)

#SAD
p.SAD <-bigdat2s %>%
  count(SAD) %>%
  mutate(prop = n/sum(n)) %>%
  ggplot(aes(x=SAD, y=n)) + 
  labs(x = "Recent Despair", y = "Number of Observations") +
  theme(legend.position = "none") +
  theme(axis.text.x = element_text(size=10, color = "black"), axis.text.y = element_text(size=10, color = "black")) +
  theme(axis.title.x = element_text(size=12, color = "black"), axis.title.y = element_text(size=10, color = "black")) +
  geom_col(aes(fill = SAD), position = "dodge",width=0.5) +
  geom_text( aes(label = scales::percent(prop), y = n), 
             size = 3,
             position = position_dodge(width = 0.9),
             vjust = 1)

#VIOLENCE
p.VIOLENCE <- bigdat2s %>%
  count(VIOLENCE) %>%
  mutate(prop = n/sum(n)) %>%
  ggplot(aes(x=VIOLENCE, y=n)) + 
  labs(x = "Recent Violence", y = "Number of Observations") +
  ylab(NULL) +
  theme(legend.position = "none") +
  theme(axis.text.x = element_text(size=10, color = "black"), axis.text.y = element_text(size=10, color = "black")) +
  theme(axis.title.x = element_text(size=12, color = "black"), axis.title.y = element_text(size=10, color = "black")) +
  geom_col(aes(fill = VIOLENCE), position = "dodge",width=0.5) +
  geom_text( aes(label = scales::percent(prop,accuracy=.11), y = n), #this is how you can round the numbers in the labels.
             size = 3,
             position = position_dodge(width = 0.9),
             vjust = 1)


#STRESS
##reorder the variable
bigdat2s$STRESS_ordered <- factor(bigdat2s$STRESS, levels=c("very high","high","some","low","none")) 

p.STRESS <- bigdat2s %>%
  count(STRESS_ordered) %>%
  mutate(prop = n/sum(n)) %>%
  ggplot(aes(x=STRESS_ordered, y=n)) + 
  geom_col(aes(fill = STRESS_ordered), position = "dodge") +
  labs(x = "Stress", y = "Number of Observations") +
  theme(legend.position = "none") +
  theme(axis.text.x = element_text(angle = 45, hjust = 1, size=10, color = "black"), axis.text.y = element_text(size=10, color = "black")) +
  theme(axis.title.x = element_text(size=12, color = "black"), axis.title.y = element_text(size=10, color = "black")) +
  geom_text( aes(label = scales::percent(prop), y = n), 
             size = 3,
             position = position_dodge(width = 0.9),
             vjust = 1)
#ES
bigdat2s$ES_ordered <- factor(bigdat2s$ES, levels=c("low","mid-low","mid","mid-high","high"))

p.ES <- bigdat2s %>%
  count(ES_ordered) %>%
  mutate(prop = n/sum(n)) %>%
  ggplot(aes(x=ES_ordered, y=n)) + 
  geom_col(aes(fill = ES_ordered), position = "dodge") +
  theme(legend.position = "none") +
  labs(x = "Family Economic Status", y = "Number of Observations") +
  theme(axis.text.x = element_text(angle = 45, hjust = 1, size=10, color = "black"), axis.text.y = element_text(size=10, color = "black")) +
  theme(axis.title.x = element_text(size=12, color = "black"), axis.title.y = element_text(size=10, color = "black")) +
  geom_text( aes(label = scales::percent(prop), y = n),
             size = 3,
             position = position_dodge(width = 0.9),
             vjust = 1)

ggarrange(p.SI, p.SAD, p.VIOLENCE, p.STRESS, p.ES,
          labels = c("a", "b", "c","d","e"),
          ncol = 3, nrow=2, font.label = list(size = 10))
```

And check their polychoric correlations (Table 1)

```
#Make a polychoric correlation table
##reorder the categorical variables appropriately
STRESS <- factor(bigdat2s$STRESS, levels=c("none", "low", "some", "high", "very high"))
ES <- factor(bigdat2s$ES, levels=c("low", "mid-low", "mid", "mid-high", "high"))

c1 <- polychor(bigdat2s$SUI_CON, bigdat2s$SAD)
c2 <- polychor(bigdat2s$SUI_CON, bigdat2s$VIOLENCE)
c3 <- polychor(bigdat2s$SUI_CON.factor, STRESS)
c4 <- polychor(bigdat2s$SUI_CON, ES)

d1 <- polychor(bigdat2s$SAD, bigdat2s$VIOLENCE)
d2 <- polychor(bigdat2s$SAD, STRESS)
d3 <- polychor(bigdat2s$SAD, ES)

e1 <- polychor(bigdat2s$VIOLENCE, STRESS)
e2 <- polychor(bigdat2s$VIOLENCE, ES)

f1 <- polychor(STRESS, ES)

cor.matrix <- cbind( c(1, c1, c2, c3, c4),
                     c(c1, 1, d1, d2, d3),
                     c(c2, d1, 1, e1, e2),
                     c(c3, d2, e1, 1, f1),
                     c(c4, d2, e2, f1, 1)
                    )

rownames(cor.matrix) <- c("1.(DV)Suicidal Ideation","2.Despair","3.Violence","4.Stress","5.Economic Status")
colnames(cor.matrix) <- c("1","2","3","4","5")
cor.matrix[upper.tri(cor.matrix)] <- NA
cor.matrix <- round(cor.matrix, 3)
cor.matrix
```

```
##                              1      2      3      4  5
## 1.(DV)Suicidal Ideation  1.000     NA     NA     NA NA
## 2.Despair                0.698  1.000     NA     NA NA
## 3.Violence               0.352  0.286  1.000     NA NA
## 4.Stress                 0.538  0.532  0.107  1.000 NA
## 5.Economic Status       -0.124 -0.099 -0.004 -0.142  1
```

### Population-averaged models with svyglm

First, fit the models.

```
design <- svydesign(id=~SCHOOL, strata=~STRATA, weights=~W,fpc=~`_TOTAL_`, data=bigdat2s)

#Basic, no interactions
fit_no_interactions <- svyglm(SUI_CON ~  SAD + VIOLENCE + STRESS  + ES +
                                HEALTH_cont +GRADE_cont + SEX + RESIDENTIAL,
                              design=design,family=binomial("logit"))
#STRESS
##sad x str  
fit_sadxstr <- svyglm(SUI_CON ~ SAD*STRESS + ES + VIOLENCE +
                        HEALTH_cont + GRADE_cont + SEX + RESIDENTIAL,
                      design=design,family=binomial("logit"))
##viol x str
fit_violxstr <- svyglm(SUI_CON ~ VIOLENCE*STRESS + ES +  SAD +
                         HEALTH_cont + GRADE_cont + SEX + RESIDENTIAL,
                       design=design,family=binomial("logit"))
#Economic Status
##sad x ES  
fit_sadxes <- svyglm(SUI_CON ~ SAD*ES + STRESS + VIOLENCE +
                       HEALTH_cont + GRADE_cont + SEX + RESIDENTIAL,
                     design=design,family=binomial("logit"))

##viol x ES 
fit_violxes <- svyglm(SUI_CON ~ VIOLENCE*ES + STRESS +SAD +
                        HEALTH_cont + GRADE_cont + SEX + RESIDENTIAL,
                      design=design,family=binomial("logit"))
```

Visualize the coefficients (S1 Fig)

```
#stress x sad
d1 <- data.frame(
  STRESS= c("b_very high", "b_high", "some", "b_low", "b_none"),
  Coef=c(coef(fit_sadxstr)[21], coef(fit_sadxstr)[20], 0, coef(fit_sadxstr)[19], coef(fit_sadxstr)[18]  ),
  SE=c(sqrt(vcov(fit_sadxstr)[21,21]), sqrt(vcov(fit_sadxstr)[20,20]), NA, sqrt(vcov(fit_sadxstr)[19,19]), sqrt(vcov(fit_sadxstr)[18,18]))
)
d1$STRESS <- factor( d1$STRESS, levels = c("b_very high", "b_high", "some", "b_low", "b_none")  )

plot_d1 <- d1 %>% 
  ggplot(aes(x = STRESS, 
             y = Coef, 
             ymin= Coef - 1.645*SE,
             ymax= Coef + 1.645*SE)) +
  geom_point(size=2.5) +
  geom_errorbar(width = 0.4, size=1) +
  geom_hline(aes(yintercept=0), colour="black", linetype="dashed") +
  labs(x = "Stress",
       y = "Interaction Coefficient with Despair") +
  theme_minimal() +
  ggtitle("Model 2") +
  theme(plot.title = element_text(colour="black", size=11.5),
        axis.title.x = element_text(face="plain",colour="black", size=11.5), axis.title.y = element_text(face="plain",colour="black", size=10),
        axis.text.x=element_text(size=10, color = "black", angle=45), axis.text.y=element_text(size=10, color = "black"))

#stress x violence
d2 <- data.frame(
  STRESS= c("b_very high", "b_high", "some", "b_low", "b_none"),
  Coef=c(coef(fit_violxstr)[21], coef(fit_violxstr)[20], 0, coef(fit_violxstr)[19], coef(fit_violxstr)[18]  ),
  SE=c(sqrt(vcov(fit_violxstr)[21,21]), sqrt(vcov(fit_violxstr)[20,20]), NA, sqrt(vcov(fit_violxstr)[19,19]), sqrt(vcov(fit_violxstr)[18,18]))
)
d2$STRESS <- factor( d2$STRESS, levels = c("b_very high", "b_high", "some", "b_low", "b_none")  )

plot_d2 <- d2 %>% 
  ggplot(aes(x = STRESS, 
             y = Coef, 
             ymin= Coef - 1.645*SE,
             ymax= Coef + 1.645*SE)) +
  geom_point(size=2.5) +
  geom_errorbar(width = 0.4, size=1) +
  geom_hline(aes(yintercept=0), colour="black", linetype="dashed") +
  labs(x = "Stress",
       y = "Interaction Coefficient with Violence") +
  theme_minimal() +
  ggtitle("Model 3") +
  theme(plot.title = element_text(colour="black", size=11.5),
        axis.title.x = element_text(face="plain",colour="black", size=11.5), axis.title.y = element_text(face="plain",colour="black", size=10),
        axis.text.x=element_text(size=10, color = "black", angle=45), axis.text.y=element_text(size=10, color = "black"))

#ecoomic status x sad
d3 <- data.frame(
  ES= c("b_low", "b_mid-low", "mid", "b_mid-high", "b_high"),
  Coef=c(coef(fit_sadxes)[18], coef(fit_sadxes)[19], 0, coef(fit_sadxes)[20], coef(fit_sadxes)[21]  ),
  SE=c(sqrt(vcov(fit_sadxes)[18,18]), sqrt(vcov(fit_sadxes)[19,19]), NA, sqrt(vcov(fit_sadxes)[20,20]), sqrt(vcov(fit_sadxes)[21,21]))
)
d3$ES <- factor( d3$ES, levels = c("b_low", "b_mid-low", "mid", "b_mid-high", "b_high")  )

plot_d3 <- d3 %>% 
  ggplot(aes(x = ES, 
             y = Coef, 
             ymin= Coef - 1.645*SE,
             ymax= Coef + 1.645*SE)) +
  geom_point(size=2.5) +
  geom_errorbar(width = 0.4, size=1) +
  geom_hline(aes(yintercept=0), colour="black", linetype="dashed") +
  labs(x = "Economic Status",
       y = "Interaction Coefficient with Despair") +
  theme_minimal() +
  ggtitle("Model 4") +
  theme(plot.title = element_text(colour="black", size=11.5),
        axis.title.x = element_text(face="plain",colour="black", size=11.5), axis.title.y = element_text(face="plain",colour="black", size=10),
        axis.text.x=element_text(size=10, color = "black", angle=45), axis.text.y=element_text(size=10, color = "black"))

#compute the p-value of the difference between b_low and b_mid-low (interaction coefficients)
diff <- coef(fit_sadxes)[18] - coef(fit_sadxes)[19]
se <- sqrt(   vcov(fit_sadxes)[18,18] + vcov(fit_sadxes)[19,19] - 2*vcov(fit_sadxes)[18,19]   )
z <- diff/se
(p <- 2*pnorm(-abs(z))) #0.03
```

```
## SAD1:ESlow 
## 0.03004814
```

```
#economic status x violence
d4 <- data.frame(
  ES= c("b_low", "b_mid-low", "mid", "b_mid-high", "b_high"),
  Coef=c(coef(fit_violxes)[18], coef(fit_violxes)[19], 0, coef(fit_violxes)[20], coef(fit_violxes)[21]  ),
  SE=c(sqrt(vcov(fit_violxes)[18,18]), sqrt(vcov(fit_violxes)[19,19]), NA, sqrt(vcov(fit_violxes)[20,20]), sqrt(vcov(fit_violxes)[21,21]))
)
d4$ES <- factor( d4$ES, levels = c("b_low", "b_mid-low", "mid", "b_mid-high", "b_high")  )

plot_d4 <- d4 %>% 
  ggplot(aes(x = ES, 
             y = Coef, 
             ymin= Coef - 1.645*SE,
             ymax= Coef + 1.645*SE)) +
  geom_point(size=2.5) +
  geom_errorbar(width = 0.4, size=1) +
  geom_hline(aes(yintercept=0), colour="black", linetype="dashed") +
  labs(x = "Economic Status",
       y = "Interaction Coefficient with Violence") +
  theme_minimal() +
  ggtitle("Model 5") +
  theme(plot.title = element_text(colour="black", size=11.5),
        axis.title.x = element_text(face="plain",colour="black", size=11.5), axis.title.y = element_text(face="plain",colour="black", size=10),
        axis.text.x=element_text(size=10, color = "black", angle=45), axis.text.y=element_text(size=10, color = "black"))

#compute the p-value the difference between b_low and b_mid-low (interaction coefficients)
diff <- coef(fit_violxes)[18] - coef(fit_violxes)[19]
se <- sqrt(   vcov(fit_violxes)[18,18] + vcov(fit_violxes)[19,19] - 2*vcov(fit_violxes)[18,19]   )
z <- diff/se
(p <- 2*pnorm(-abs(z))) #0.103
```

```
## VIOLENCE1:ESlow 
##       0.1029997
```

```
#compute the p-value of the difference between b_mig-high and b_high (interaction coefficients)
diff <- coef(fit_violxes)[21] - coef(fit_violxes)[20]
se <- sqrt(   vcov(fit_violxes)[21,21] + vcov(fit_violxes)[20,20] - 2*vcov(fit_violxes)[21,20]   )
z <- diff/se
(p <- 2*pnorm(-abs(z))) #0.016
```

```
## VIOLENCE1:EShigh 
##       0.01647026
```

```
(
  coefplot <- ggarrange(plot_d1, plot_d2, plot_d3, plot_d4,
                      labels = c("a", "b", "c", "d"),
                      ncol = 4, nrow=1, 
                      font.label = list(size = 11.5))
)
```

Next, plot the predicted probabilities (Fig 2)

```
#stress x sad
newdat.s1 <- data.frame(
  "SAD"= c( rep("0",5),  rep("1",5)   ),
  "STRESS"= c(rep(  c("very high","high", "some", "low", "none"),2) ),
  "HEALTH_cont"= c(rep(0,10) ), 
  "ES"= c(rep("mid",10) ),
  "VIOLENCE" = c( rep("0",10) ),
  "SEX" = c( rep(0,10) ),
  "GRADE_cont" = c(rep(0, 10)),
  "RESIDENTIAL" = c(rep("family", 10))
  )
newdat.s1

pred <- predict(fit_sadxstr, newdata = newdat.s1, type="link")
df <- data.frame( "DESPAIR" = c(rep("0",5), rep("1",5)),
                  "STRESS" = c( "very high","high","some","low","none", "very high","high","some","low","none")
)
dats1 <- cbind(df, pred)
dats1$STRESS <- factor( dats1$STRESS, levels = c("very high","high","some","low","none")  )
dats1 #the 'link' column shows the logit values, the SE column the logit SEs

graphs1 <- dats1 %>% 
  ggplot(aes(x = STRESS, 
             y = logistic(link), 
             color = DESPAIR,
             ymin=logistic(link - 1.645*SE),
             ymax=logistic(link + 1.645*SE))) +
  geom_point(size=2.5) +
  geom_errorbar(width = 0.4, size=1) +
  ylim(0, 0.5) +
  labs(x = "Stress",
       color  = "Despair/Grief",
       y = "Probability of Suicidal Ideation") +
  theme_minimal() +
  ggtitle("Model 2") +
  theme(plot.title = element_text(colour="black", size=11.5),
        legend.text = element_text(colour="black",size=10), legend.title = element_text(face="plain",colour="black",     size=10),legend.position="bottom",
        axis.title.x = element_text(face="plain",colour="black", size=11.5), axis.title.y = element_text(face="plain",colour="black", size=10),
        axis.text.x=element_text(size=10, color = "black", angle=45), axis.text.y=element_text(size=10, color = "black")) +
  scale_color_manual(labels = c("0"= "No", "1"=  "Yes"), values=c("blue","red"))


#stress x violence
newdat.v1 <- data.frame(
  "VIOLENCE"= c( rep("0",5),  rep("1",5)   ),
  "STRESS"= c(rep(  c("very high","high", "some", "low", "none"),2) ),
  "HEALTH_cont"= c(rep(0,10) ), 
  "ES"= c(rep("mid",10) ),
  "SAD" = c( rep("0",10) ),
  "SEX" = c( rep(0,10) ),
  "GRADE_cont" = c(rep(0, 10)),
  "RESIDENTIAL" = c(rep("family", 10))
  )

pred <- predict(fit_violxstr, newdata = newdat.v1, type="link")
df <- data.frame( "VIOLENCE" = c(rep("0",5), rep("1",5)),
                  "STRESS" = c( "very high","high","some","low","none", "very high","high","some","low","none")
)
datv1 <- cbind(df, pred)
datv1$STRESS <- factor( datv1$STRESS, levels = c("very high","high","some","low","none")  )

graphv1 <- datv1 %>% 
  ggplot(aes(x = STRESS, 
             y = logistic(link), 
             color = VIOLENCE,
             ymin=logistic(link - 1.645*SE),
             ymax=logistic(link + 1.645*SE))) +
  geom_point(size=2.5) +
  geom_errorbar(width = 0.4, size=1) +
  ylim(0, 0.25) +
  labs(x = "Stress",
       color  = "Violence",
       y = "Probability of Suicidal Ideation") +
  theme_minimal() +
  ggtitle("Model 3") +
  theme(plot.title = element_text(colour="black", size=11.5),
        legend.text = element_text(colour="black",size=10), legend.title = element_text(face="plain",colour="black", size=10),legend.position="bottom",
        axis.title.x = element_text(face="plain",colour="black", size=11.5), axis.title.y = element_text(face="plain",colour="black", size=10),
        axis.text.x=element_text(size=10, color = "black", angle=45), axis.text.y=element_text(size=10, color = "black")) +
  scale_color_manual(labels = c("0"= "No", "1"=  "Yes"), values=c("blue","red"))


#economic status x sad
newdat.s2 <- data.frame(
  "SAD"= c( rep("0",5),  rep("1",5)   ),
  "ES"= c( rep(  c("low","mid-low", "mid", "mid-high", "high"),2) ),
  "STRESS"= c(rep("some",10) ), 
  "HEALTH_cont"= c(rep(0,10) ),
  "VIOLENCE" = c( rep("0",10) ),
  "SEX" = c( rep(0,10) ),
  "GRADE_cont" = c(rep(0, 10)),
  "RESIDENTIAL" = c(rep("family", 10))
  )

pred <- predict(fit_sadxes, newdata = newdat.s2, type="link")
df <- data.frame( "DESPAIR" = c(rep("0",5), rep("1",5)),
                  "ES" = c( "low","mid-low", "mid", "mid-high", "high", "low","mid-low", "mid", "mid-high", "high")
)
dats2 <- cbind(df, pred)
dats2$ES <- factor( dats2$ES, levels = c("low","mid-low", "mid", "mid-high", "high")  )


graphs2 <- dats2 %>% 
  ggplot(aes(x = ES, 
             y = logistic(link), 
             color = DESPAIR,
             ymin=logistic(link - 1.645*SE),
             ymax=logistic(link + 1.645*SE))) +
  geom_point(size=2.5) +
  geom_errorbar(width = 0.4, size=1) +
  ylim(0, 0.25) +
  labs(x = "Economic Status",
       color  = "Despair/Grief",
       y = "Probability of Suicidal Ideation") +
  theme_minimal() +
  ggtitle("Model 4") +
  theme(plot.title = element_text(colour="black", size=11.5),
        legend.text = element_text(colour="black",size=10), legend.title = element_text(face="plain",colour="black", size=10),legend.position="bottom",
        axis.title.x = element_text(face="plain",colour="black", size=11.5), axis.title.y = element_text(face="plain",colour="black", size=10),
        axis.text.x=element_text(size=10, color = "black", angle=45), axis.text.y=element_text(size=10, color = "black")) +
  scale_color_manual(labels = c("0"= "No", "1"=  "Yes"), values=c("#00AFBB","#FC4E07"))


#economic status x violence
newdat.v2 <- data.frame(
  "VIOLENCE"= c( rep("0",5),  rep("1",5)   ),
  "ES"= c( rep(  c("low","mid-low", "mid", "mid-high", "high"),2) ),
  "STRESS"= c(rep("some",10) ), 
  "HEALTH_cont"= c(rep(0,10) ), 
  "SAD" = c( rep("0",10) ),
  "SEX" = c( rep(0,10) ),
  "GRADE_cont" = c(rep(0, 10)),
  "RESIDENTIAL" = c(rep("family", 10))
  )
newdat.v2

pred <- predict(fit_violxes, newdata = newdat.v2, type="link")
df <- data.frame( "VIOLENCE" = c(rep("0",5), rep("1",5)),
                  "ES" = c( "low","mid-low", "mid", "mid-high", "high", "low","mid-low", "mid", "mid-high", "high")
)
datv2 <- cbind(df, pred)
datv2$ES <- factor( datv2$ES, levels = c("low","mid-low", "mid", "mid-high", "high")  )

graphv2 <- datv2 %>% 
  ggplot(aes(x = ES, 
             y = logistic(link), 
             color = VIOLENCE,
             ymin=logistic(link - 1.645*SE),
             ymax=logistic(link + 1.645*SE))) +
  geom_point(size=2.5) +
  geom_errorbar(width = 0.4, size=1) +
  ylim(0, 0.2) +
  labs(x = "Economic Status",
       color  = "Violence",
       y = "Probability of Suicidal Ideation") +
  theme_minimal() +
  ggtitle("Model 5") +
  theme(plot.title = element_text(colour="black", size=11.5),
        legend.text = element_text(colour="black",size=10), legend.title = element_text(face="plain",colour="black", size=10),legend.position="bottom",
        axis.title.x = element_text(face="plain",colour="black", size=11.5), axis.title.y = element_text(face="plain",colour="black", size=10),
        axis.text.x=element_text(size=10, color = "black", angle=45), axis.text.y=element_text(size=10, color = "black")) +
  scale_color_manual(labels = c("0"= "No", "1"=  "Yes"), values=c("#00AFBB","#FC4E07"))


g1 <- ggarrange(graphs1, graphv1,
          labels = c("a", "b"),
          ncol = 2, nrow=1, font.label = list(size = 11.5), legend="top")

g2 <- ggarrange(graphs2, graphv2,
                labels = c("c", "d"),
                ncol = 2, nrow=1, font.label = list(size = 11.5), legend="top")
```

```
(g3 <-ggarrange(g1, g2, ncol=2, nrow=1))
```

Make a combined regression table (Table 1, not run in this .html presentation)

```
stargazer(fit_nointeractions, fit_sadxstr, fit_violxstr, fit_sadxes, fit_violxes,
          ci=T, omit.stat = c("ll"), digits=2, model.numbers = T, type="html",
          star.cutoffs = c(0.1, 0.05, 0.01, 0.001), star.char = c("+","*","**","***"),notes.append = F,
          notes="+ p<0.1; * p<0.05; ** p<0.01; *** p<0.001",
          out="svyglm Table.htm")
```

Next, plot the risk ratio (Fig 3). The standard error will be approximated using the delta method.

```
# SAD - STRESS none
pred1 <- predict(fit_sadxstr,
                 newdata = data.frame(SAD="1", STRESS="none", ES="mid",
                                      HEALTH_cont=0, VIOLENCE="0",
                                      GRADE_cont=0, SEX=0, RESIDENTIAL="family"),
                 type="response")
pred0 <- predict(fit_sadxstr,
                 newdata = data.frame(SAD="0", STRESS="none", ES="mid",
                                      HEALTH_cont=0, VIOLENCE="0",
                                      GRADE_cont=0, SEX=0, RESIDENTIAL="family"),
                 type="response")
(RR_sad_str_none <-(pred1/pred0)[1])
betas <- as.vector(coef(fit_sadxstr))

se_sad_str_none <- deltamethod( ~(1 + exp(-x1 -0*x2 -1*x3 -0*x4 - 0*x5 -0*x6 - 0*x7 - 0*x8 - 0*x9 - 0*x10 - 0*x11 - 0*x12 -0*x13 -0*x14 -0*x15 -0*x16 - 0*x17 -0*x18 - 0*x19 -0*x20 - 0*x21)) /
                                 (1 + exp(-x1 -1*x2 -1*x3 -0*x4 - 0*x5 -0*x6 - 0*x7 - 0*x8 - 0*x9 - 0*x10 - 0*x11 - 0*x12 -0*x13 -0*x14 -0*x15 -0*x16 - 0*x17 -1*x18 - 0*x19 -0*x20 - 0*x21)), 
                              betas, vcov(fit_sadxstr) 
); se_sad_str_none


#SAD - STRESS low
pred1 <- predict(fit_sadxstr,
                 newdata = data.frame(SAD="1", STRESS="low", ES="mid",
                                      HEALTH_cont=0, VIOLENCE="0",
                                      GRADE_cont=0, SEX=0, RESIDENTIAL="family"),
                 type="response")
pred0 <- predict(fit_sadxstr,
                 newdata = data.frame(SAD="0", STRESS="low", ES="mid",
                                      HEALTH_cont=0, VIOLENCE="0",
                                      GRADE_cont=0, SEX=0, RESIDENTIAL="family"),
                 type="response")
(RR_sad_str_low <-(pred1/pred0)[1])
betas <- as.vector(coef(fit_sadxstr))
se_sad_str_low <- deltamethod( ~(1 + exp(-x1 -0*x2 -0*x3 -1*x4 - 0*x5 -0*x6 - 0*x7 - 0*x8 - 0*x9 - 0*x10 - 0*x11 - 0*x12 -0*x13 -0*x14 -0*x15 -0*x16 - 0*x17 -0*x18 - 0*x19 -0*x20 - 0*x21)) /
                                (1 + exp(-x1 -1*x2 -0*x3 -1*x4 - 0*x5 -0*x6 - 0*x7 - 0*x8 - 0*x9 - 0*x10 - 0*x11 - 0*x12 -0*x13 -0*x14 -0*x15 -0*x16 - 0*x17 -0*x18 - 1*x19 -0*x20 - 0*x21)), 
                                betas, vcov(fit_sadxstr) 
); se_sad_str_low


#SAD - STRESS some
pred1 <- predict(fit_sadxstr,
                 newdata = data.frame(SAD="1", STRESS="some", ES="mid",
                                      HEALTH_cont=0, VIOLENCE="0",
                                      GRADE_cont=0, SEX=0, RESIDENTIAL="family"),
                 type="response")
pred0 <- predict(fit_sadxstr,
                 newdata = data.frame(SAD="0", STRESS="some", ES="mid",
                                      HEALTH_cont=0, VIOLENCE="0",
                                      GRADE_cont=0, SEX=0, RESIDENTIAL="family"),
                 type="response")
(RR_sad_str_some <-(pred1/pred0)[1])
betas <- as.vector(coef(fit_sadxstr))
se_sad_str_some <- deltamethod( ~(1 + exp(-x1 -0*x2 -0*x3 -0*x4 - 0*x5 -0*x6 - 0*x7 - 0*x8 - 0*x9 - 0*x10 - 0*x11 - 0*x12 -0*x13 -0*x14 -0*x15 -0*x16 - 0*x17 -0*x18 - 0*x19 -0*x20 - 0*x21)) /
                                 (1 + exp(-x1 -1*x2 -0*x3 -0*x4 - 0*x5 -0*x6 - 0*x7 - 0*x8 - 0*x9 - 0*x10 - 0*x11 - 0*x12 -0*x13 -0*x14 -0*x15 -0*x16 - 0*x17 -0*x18 - 0*x19 -0*x20 - 0*x21)), 
                               betas, vcov(fit_sadxstr) 
); se_sad_str_some


#SAD - STRESS high
pred1 <- predict(fit_sadxstr,
                 newdata = data.frame(SAD="1", STRESS="high", ES="mid",
                                      HEALTH_cont=0, VIOLENCE="0",
                                      GRADE_cont=0, SEX=0, RESIDENTIAL="family"),
                 type="response")
pred0 <- predict(fit_sadxstr,
                 newdata = data.frame(SAD="0", STRESS="high", ES="mid",
                                      HEALTH_cont=0, VIOLENCE="0",
                                      GRADE_cont=0, SEX=0, RESIDENTIAL="family"),
                 type="response")
(RR_sad_str_high <-(pred1/pred0)[1])
betas <- as.vector(coef(fit_sadxstr))
se_sad_str_high <- deltamethod( ~(1 + exp(-x1 -0*x2 -0*x3 -0*x4 - 1*x5 -0*x6 - 0*x7 - 0*x8 - 0*x9 - 0*x10 - 0*x11 - 0*x12 -0*x13 -0*x14 -0*x15 -0*x16 - 0*x17 -0*x18 - 0*x19 -0*x20 - 0*x21)) /
                                 (1 + exp(-x1 -1*x2 -0*x3 -0*x4 - 1*x5 -0*x6 - 0*x7 - 0*x8 - 0*x9 - 0*x10 - 0*x11 - 0*x12 -0*x13 -0*x14 -0*x15 -0*x16 - 0*x17 -0*x18 - 0*x19 -1*x20 - 0*x21)), 
                                betas, vcov(fit_sadxstr) 
); se_sad_str_high


#SAD - STRESS very high
pred1 <- predict(fit_sadxstr,
                 newdata = data.frame(SAD="1", STRESS="very high", ES="mid",
                                      HEALTH_cont=0, VIOLENCE="0",
                                      GRADE_cont=0, SEX=0, RESIDENTIAL="family"),
                 type="response")
pred0 <- predict(fit_sadxstr,
                 newdata = data.frame(SAD="0", STRESS="very high", ES="mid",
                                      HEALTH_cont=0, VIOLENCE="0",
                                      GRADE_cont=0, SEX=0, RESIDENTIAL="family"),
                 type="response")
(RR_sad_str_vhigh <-(pred1/pred0)[1])
betas <- as.vector(coef(fit_sadxstr))
se_sad_str_vhigh <- deltamethod( ~(1 + exp(-x1 -0*x2 -0*x3 -0*x4 - 0*x5 -1*x6 - 0*x7 - 0*x8 - 0*x9 - 0*x10 - 0*x11 - 0*x12 -0*x13 -0*x14 -0*x15 -0*x16 - 0*x17 -0*x18 - 0*x19 -0*x20 - 0*x21)) /
                                  (1 + exp(-x1 -1*x2 -0*x3 -0*x4 - 0*x5 -1*x6 - 0*x7 - 0*x8 - 0*x9 - 0*x10 - 0*x11 - 0*x12 -0*x13 -0*x14 -0*x15 -0*x16 - 0*x17 -0*x18 - 0*x19 -0*x20 - 1*x21)), 
                                betas, vcov(fit_sadxstr) 
); se_sad_str_vhigh


df_sad_str <- data.frame(
           STRESS= c("very high", "high", "some", "low", "none"),
           RR=c(RR_sad_str_vhigh, RR_sad_str_high, RR_sad_str_some, RR_sad_str_low, RR_sad_str_none),
           Lower=c(RR_sad_str_vhigh - 1.645*se_sad_str_vhigh, RR_sad_str_high - 1.645*se_sad_str_high,
                   RR_sad_str_some - 1.645*se_sad_str_some, RR_sad_str_low - 1.645*se_sad_str_low, RR_sad_str_none - 1.645*se_sad_str_none),
           Upper=c(RR_sad_str_vhigh + 1.645*se_sad_str_vhigh, RR_sad_str_high + 1.645*se_sad_str_high,
                   RR_sad_str_some + 1.645*se_sad_str_some, RR_sad_str_low + 1.645*se_sad_str_low, RR_sad_str_none + 1.645*se_sad_str_none)
)

df_sad_str$STRESS <- factor( df_sad_str$STRESS, levels = c("very high","high","some","low","none")  )

plot_sad_str <- df_sad_str %>% 
  ggplot(aes(x = STRESS, 
             y = RR, 
             ymin=Lower,
             ymax=Upper)) +
  geom_point(size=2.5) +
  geom_errorbar(width = 0.4, size=1) +
  #ylim(0, 0.5) +
  labs(x = "Stress",
       y = "Conditional Relative Risk") +
  theme_minimal() +
  ggtitle("Model 2") +
  theme(plot.title = element_text(colour="black", size=11.5),
        axis.title.x = element_text(face="plain",colour="black", size=11.5), axis.title.y = element_text(face="plain",colour="black", size=10),
        axis.text.x=element_text(size=10, color = "black", angle=45), axis.text.y=element_text(size=10, color = "black"))
  


# VIOLENCE - STRESS none
pred1 <- predict(fit_violxstr,
                 newdata = data.frame(VIOLENCE="1", STRESS="none", ES="mid",
                                      HEALTH_cont=0, SAD="0",
                                      GRADE_cont=0, SEX=0, RESIDENTIAL="family"),
                 type="response")
pred0 <- predict(fit_violxstr,
                 newdata = data.frame(VIOLENCE="0", STRESS="none", ES="mid",
                                      HEALTH_cont=0, SAD="0",
                                      GRADE_cont=0, SEX=0, RESIDENTIAL="family"),
                 type="response")
(RR_viol_str_none <-(pred1/pred0)[1])
betas <- as.vector(coef(fit_violxstr))
se_viol_str_none <- deltamethod( ~(1 + exp(-x1 -0*x2 -1*x3 -0*x4 - 0*x5 -0*x6 - 0*x7 - 0*x8 - 0*x9 - 0*x10 - 0*x11 - 0*x12 -0*x13 -0*x14 -0*x15 -0*x16 - 0*x17 -0*x18 - 0*x19 -0*x20 - 0*x21)) /
                                  (1 + exp(-x1 -1*x2 -1*x3 -0*x4 - 0*x5 -0*x6 - 0*x7 - 0*x8 - 0*x9 - 0*x10 - 0*x11 - 0*x12 -0*x13 -0*x14 -0*x15 -0*x16 - 0*x17 -1*x18 - 0*x19 -0*x20 - 0*x21)), 
                                betas, vcov(fit_sadxstr) 
); se_viol_str_none


#viol - STRESS low
pred1 <- predict(fit_violxstr,
                 newdata = data.frame(VIOLENCE="1", STRESS="low", ES="mid",
                                      HEALTH_cont=0, SAD="0",
                                      GRADE_cont=0, SEX=0, RESIDENTIAL="family"),
                 type="response")
pred0 <- predict(fit_violxstr,
                 newdata = data.frame(VIOLENCE="0", STRESS="low", ES="mid",
                                      HEALTH_cont=0, SAD="0",
                                      GRADE_cont=0, SEX=0, RESIDENTIAL="family"),
                 type="response")
(RR_viol_str_low <-(pred1/pred0)[1])
betas <- as.vector(coef(fit_violxstr))
se_viol_str_low <- deltamethod( ~(1 + exp(-x1 -0*x2 -0*x3 -1*x4 - 0*x5 -0*x6 - 0*x7 - 0*x8 - 0*x9 - 0*x10 - 0*x11 - 0*x12 -0*x13 -0*x14 -0*x15 -0*x16 - 0*x17 -0*x18 - 0*x19 -0*x20 - 0*x21)) /
                                  (1 + exp(-x1 -1*x2 -0*x3 -1*x4 - 0*x5 -0*x6 - 0*x7 - 0*x8 - 0*x9 - 0*x10 - 0*x11 - 0*x12 -0*x13 -0*x14 -0*x15 -0*x16 - 0*x17 -0*x18 - 1*x19 -0*x20 - 0*x21)), 
                                betas, vcov(fit_violxstr) 
); se_viol_str_low


#viol - STRESS some
pred1 <- predict(fit_violxstr,
                 newdata = data.frame(VIOLENCE="1", STRESS="some", ES="mid",
                                      HEALTH_cont=0, SAD="0",
                                      GRADE_cont=0, SEX=0, RESIDENTIAL="family"),
                 type="response")
pred0 <- predict(fit_violxstr,
                 newdata = data.frame(VIOLENCE="0", STRESS="some", ES="mid",
                                      HEALTH_cont=0, SAD="0",
                                      GRADE_cont=0, SEX=0, RESIDENTIAL="family"),
                 type="response")
(RR_viol_str_some <-(pred1/pred0)[1])
betas <- as.vector(coef(fit_violxstr))
se_viol_str_some <- deltamethod( ~(1 + exp(-x1 -0*x2 -0*x3 -0*x4 - 0*x5 -0*x6 - 0*x7 - 0*x8 - 0*x9 - 0*x10 - 0*x11 - 0*x12 -0*x13 -0*x14 -0*x15 -0*x16 - 0*x17 -0*x18 - 0*x19 -0*x20 - 0*x21)) /
                                   (1 + exp(-x1 -1*x2 -0*x3 -0*x4 - 0*x5 -0*x6 - 0*x7 - 0*x8 - 0*x9 - 0*x10 - 0*x11 - 0*x12 -0*x13 -0*x14 -0*x15 -0*x16 - 0*x17 -0*x18 - 0*x19 -0*x20 - 0*x21)), 
                                 betas, vcov(fit_violxstr) 
); se_viol_str_some


#viol - STRESS high
pred1 <- predict(fit_violxstr,
                 newdata = data.frame(VIOLENCE="1", 
                                      STRESS="high", ES="mid", HEALTH_cont=0, SAD="0",
                                      GRADE_cont=0, SEX=0, RESIDENTIAL="family"),
                 type="response")
pred0 <- predict(fit_violxstr,
                 newdata = data.frame(VIOLENCE="0", 
                                      STRESS="high", ES="mid", HEALTH_cont=0, SAD="0",
                                      GRADE_cont=0, SEX=0, RESIDENTIAL="family"),
                 type="response")
(RR_viol_str_high <-(pred1/pred0)[1])
betas <- as.vector(coef(fit_violxstr))
se_viol_str_high <- deltamethod( ~(1 + exp(-x1 -0*x2 -0*x3 -0*x4 - 1*x5 -0*x6 - 0*x7 - 0*x8 - 0*x9 - 0*x10 - 0*x11 - 0*x12 -0*x13 -0*x14 -0*x15 -0*x16 - 0*x17 -0*x18 - 0*x19 -0*x20 - 0*x21)) /
                                   (1 + exp(-x1 -1*x2 -0*x3 -0*x4 - 1*x5 -0*x6 - 0*x7 - 0*x8 - 0*x9 - 0*x10 - 0*x11 - 0*x12 -0*x13 -0*x14 -0*x15 -0*x16 - 0*x17 -0*x18 - 0*x19 -1*x20 - 0*x21)), 
                                 betas, vcov(fit_violxstr) 
); se_viol_str_high


#viol - STRESS very high
pred1 <- predict(fit_violxstr,
                 newdata = data.frame(VIOLENCE="1", 
                                      STRESS="very high", ES="mid", HEALTH_cont=0, SAD="0",
                                      GRADE_cont=0, SEX=0, RESIDENTIAL="family"),
                 type="response")
pred0 <- predict(fit_violxstr,
                 newdata = data.frame(VIOLENCE="0",
                                      STRESS="very high", ES="mid", HEALTH_cont=0, SAD="0",
                                      GRADE_cont=0, SEX=0, RESIDENTIAL="family"),
                 type="response")
(RR_viol_str_vhigh <-(pred1/pred0)[1])
betas <- as.vector(coef(fit_violxstr))
se_viol_str_vhigh <- deltamethod( ~(1 + exp(-x1 -0*x2 -0*x3 -0*x4 - 0*x5 -1*x6 - 0*x7 - 0*x8 - 0*x9 - 0*x10 - 0*x11 - 0*x12 -0*x13 -0*x14 -0*x15 -0*x16 - 0*x17 -0*x18 - 0*x19 -0*x20 - 0*x21)) /
                                    (1 + exp(-x1 -1*x2 -0*x3 -0*x4 - 0*x5 -1*x6 - 0*x7 - 0*x8 - 0*x9 - 0*x10 - 0*x11 - 0*x12 -0*x13 -0*x14 -0*x15 -0*x16 - 0*x17 -0*x18 - 0*x19 -0*x20 - 1*x21)), 
                                  betas, vcov(fit_violxstr) 
); se_viol_str_vhigh


df_viol_str <- data.frame(           
                          STRESS= c("very high", "high", "some", "low", "none"),
                          RR= c(RR_viol_str_vhigh, RR_viol_str_high, RR_viol_str_some, RR_viol_str_low, RR_viol_str_none),
                          Lower= c(RR_viol_str_vhigh - 1.645*se_viol_str_vhigh, RR_viol_str_high - 1.645*se_viol_str_high,
                                  RR_viol_str_some - 1.645*se_viol_str_some, RR_viol_str_low - 1.645*se_viol_str_low, RR_viol_str_none - 1.645*se_viol_str_none),
                          Upper= c(RR_viol_str_vhigh + 1.645*se_viol_str_vhigh, RR_viol_str_high + 1.645*se_viol_str_high,
                                  RR_viol_str_some + 1.645*se_viol_str_some, RR_viol_str_low + 1.645*se_viol_str_low, RR_viol_str_none + 1.645*se_viol_str_none)
)

df_viol_str$STRESS <- factor( df_viol_str$STRESS, levels = c("very high","high","some","low","none")  )

plot_viol_str <- df_viol_str %>% 
  ggplot(aes(x = STRESS, 
             y = RR, 
             ymin=Lower,
             ymax=Upper)) +
  geom_point(size=2.5) +
  geom_errorbar(width = 0.4, size=1) +
  #ylim(0, 0.5) +
  labs(x = "Stress",
       y = "Conditional Relative Risk") +
  theme_minimal() +
  ggtitle("Model 3") +
  theme(plot.title = element_text(colour="black", size=11.5),
        axis.title.x = element_text(face="plain",colour="black", size=11.5), axis.title.y = element_text(face="plain",colour="black", size=10),
        axis.text.x=element_text(size=10, color = "black", angle=45), axis.text.y=element_text(size=10, color = "black"))


# SAD - ES low

pred1 <- predict(fit_sadxes,
                 newdata = data.frame(SAD="1", ES="low", STRESS="some",
                                      HEALTH_cont=0, VIOLENCE="0",
                                      GRADE_cont=0, SEX=0, RESIDENTIAL="family"),
                 type="response")
pred0 <- predict(fit_sadxes,
                 newdata = data.frame(SAD="0", ES="low", STRESS="some",
                                      HEALTH_cont=0, VIOLENCE="0",
                                      GRADE_cont=0, SEX=0, RESIDENTIAL="family"),
                 type="response")
(RR_sad_es_low <-(pred1/pred0)[1])
betas <- as.vector(coef(fit_sadxes))
se_sad_es_low <- deltamethod( ~(1 + exp(-x1 -0*x2 -1*x3 -0*x4 - 0*x5 -0*x6 - 0*x7 - 0*x8 - 0*x9 - 0*x10 - 0*x11 - 0*x12 -0*x13 -0*x14 -0*x15 -0*x16 - 0*x17 -0*x18 - 0*x19 -0*x20 - 0*x21)) /
                                (1 + exp(-x1 -1*x2 -1*x3 -0*x4 - 0*x5 -0*x6 - 0*x7 - 0*x8 - 0*x9 - 0*x10 - 0*x11 - 0*x12 -0*x13 -0*x14 -0*x15 -0*x16 - 0*x17 -1*x18 - 0*x19 -0*x20 - 0*x21)), 
                              betas, vcov(fit_sadxes) 
); se_sad_es_low


#SAD - ES mid-low
pred1 <- predict(fit_sadxes,
                 newdata = data.frame(SAD="1", ES="mid-low", STRESS="some",
                                      HEALTH_cont=0, VIOLENCE="0",
                                      GRADE_cont=0, SEX=0, RESIDENTIAL="family"),
                 type="response")
pred0 <- predict(fit_sadxes,
                 newdata = data.frame(SAD="0", ES="mid-low", STRESS="some",
                                      HEALTH_cont=0, VIOLENCE="0",
                                      GRADE_cont=0, SEX=0, RESIDENTIAL="family"),
                 type="response")
(RR_sad_es_midlow <-(pred1/pred0)[1])
betas <- as.vector(coef(fit_sadxes))
se_sad_es_midlow <- deltamethod( ~(1 + exp(-x1 -0*x2 -0*x3 -1*x4 - 0*x5 -0*x6 - 0*x7 - 0*x8 - 0*x9 - 0*x10 - 0*x11 - 0*x12 -0*x13 -0*x14 -0*x15 -0*x16 - 0*x17 -0*x18 - 0*x19 -0*x20 - 0*x21)) /
                                   (1 + exp(-x1 -1*x2 -0*x3 -1*x4 - 0*x5 -0*x6 - 0*x7 - 0*x8 - 0*x9 - 0*x10 - 0*x11 - 0*x12 -0*x13 -0*x14 -0*x15 -0*x16 - 0*x17 -0*x18 - 1*x19 -0*x20 - 0*x21)), 
                                 betas, vcov(fit_sadxes) 
); se_sad_es_midlow


#SAD - ES mid
pred1 <- predict(fit_sadxes,
                 newdata = data.frame(SAD="1", ES="mid", STRESS="some",
                                      HEALTH_cont=0, VIOLENCE="0",
                                      GRADE_cont=0, SEX=0, RESIDENTIAL="family"),
                 type="response")
pred0 <- predict(fit_sadxes,
                 newdata = data.frame(SAD="0", ES="mid", STRESS="some",
                                      HEALTH_cont=0, VIOLENCE="0",
                                      GRADE_cont=0, SEX=0, RESIDENTIAL="family"),
                 type="response")
(RR_sad_es_mid <-(pred1/pred0)[1])
betas <- as.vector(coef(fit_sadxes))
se_sad_es_mid <- deltamethod( ~(1 + exp(-x1 -0*x2 -0*x3 -0*x4 - 0*x5 -0*x6 - 0*x7 - 0*x8 - 0*x9 - 0*x10 - 0*x11 - 0*x12 -0*x13 -0*x14 -0*x15 -0*x16 - 0*x17 -0*x18 - 0*x19 -0*x20 - 0*x21)) /
                                (1 + exp(-x1 -1*x2 -0*x3 -0*x4 - 0*x5 -0*x6 - 0*x7 - 0*x8 - 0*x9 - 0*x10 - 0*x11 - 0*x12 -0*x13 -0*x14 -0*x15 -0*x16 - 0*x17 -0*x18 - 0*x19 -0*x20 - 0*x21)), 
                              betas, vcov(fit_sadxes) 
); se_sad_es_mid


#SAD - ES mid-high
pred1 <- predict(fit_sadxes,
                 newdata = data.frame(SAD="1", ES="mid-high", STRESS="some",
                                      HEALTH_cont=0, VIOLENCE="0",
                                      GRADE_cont=0, SEX=0, RESIDENTIAL="family"),
                 type="response")
pred0 <- predict(fit_sadxes,
                 newdata = data.frame(SAD="0", ES="mid-high", STRESS="some",
                                      HEALTH_cont=0, VIOLENCE="0",
                                      GRADE_cont=0, SEX=0, RESIDENTIAL="family"),
                 type="response")
(RR_sad_es_midhigh <-(pred1/pred0)[1])
betas <- as.vector(coef(fit_sadxes))
se_sad_es_midhigh <- deltamethod( ~(1 + exp(-x1 -0*x2 -0*x3 -0*x4 - 1*x5 -0*x6 - 0*x7 - 0*x8 - 0*x9 - 0*x10 - 0*x11 - 0*x12 -0*x13 -0*x14 -0*x15 -0*x16 - 0*x17 -0*x18 - 0*x19 -0*x20 - 0*x21)) /
                                    (1 + exp(-x1 -1*x2 -0*x3 -0*x4 - 1*x5 -0*x6 - 0*x7 - 0*x8 - 0*x9 - 0*x10 - 0*x11 - 0*x12 -0*x13 -0*x14 -0*x15 -0*x16 - 0*x17 -0*x18 - 0*x19 -1*x20 - 0*x21)), 
                                  betas, vcov(fit_sadxes) 
); se_sad_es_midhigh


#SAD - ES high
pred1 <- predict(fit_sadxes,
                 newdata = data.frame(SAD="1", ES="high", STRESS="some",
                                      HEALTH_cont=0, VIOLENCE="0",
                                      GRADE_cont=0, SEX=0, RESIDENTIAL="family"),
                 type="response")
pred0 <- predict(fit_sadxes,
                 newdata = data.frame(SAD="0", ES="high", STRESS="some",
                                      HEALTH_cont=0, VIOLENCE="0",
                                      GRADE_cont=0, SEX=0, RESIDENTIAL="family"),
                 type="response")
(RR_sad_es_high <-(pred1/pred0)[1])
betas <- as.vector(coef(fit_sadxes))
se_sad_es_high <- deltamethod( ~(1 + exp(-x1 -0*x2 -0*x3 -0*x4 - 0*x5 -1*x6 - 0*x7 - 0*x8 - 0*x9 - 0*x10 - 0*x11 - 0*x12 -0*x13 -0*x14 -0*x15 -0*x16 - 0*x17 -0*x18 - 0*x19 -0*x20 - 0*x21)) /
                                 (1 + exp(-x1 -1*x2 -0*x3 -0*x4 - 0*x5 -1*x6 - 0*x7 - 0*x8 - 0*x9 - 0*x10 - 0*x11 - 0*x12 -0*x13 -0*x14 -0*x15 -0*x16 - 0*x17 -0*x18 - 0*x19 -0*x20 - 1*x21)), 
                               betas, vcov(fit_sadxes) 
); se_sad_es_high


df_sad_es <- data.frame(
                        ES=c("low", "mid-low", "mid", "mid-high", "high"),
                        RR=c(RR_sad_es_low, RR_sad_es_midlow, RR_sad_es_mid, RR_sad_es_midhigh, RR_sad_es_high),
                        Lower=c(RR_sad_es_low - 1.645*se_sad_es_low, RR_sad_es_midlow - 1.645*se_sad_es_midlow,
                                RR_sad_es_mid - 1.645*se_sad_es_mid, RR_sad_es_midhigh - 1.645*se_sad_es_midhigh, RR_sad_es_high - 1.645*se_sad_es_high),
                        Upper=c(RR_sad_es_low + 1.645*se_sad_es_low, RR_sad_es_midlow + 1.645*se_sad_es_midlow,
                                RR_sad_es_mid + 1.645*se_sad_es_mid, RR_sad_es_midhigh + 1.645*se_sad_es_midhigh, RR_sad_es_high + 1.645*se_sad_es_high)
)

df_sad_es$ES <- factor( df_sad_es$ES, levels = c("low", "mid-low", "mid", "mid-high", "high")  )

plot_sad_es <- df_sad_es %>% 
  ggplot(aes(x = ES, 
             y = RR, 
             ymin=Lower,
             ymax=Upper)) +
  geom_point(size=2.5) +
  geom_errorbar(width = 0.4, size=1) +
  #ylim(0, 0.5) +
  labs(x = "Economic Status",
       y = "Conditional Relative Risk") +
  theme_minimal() +
  ggtitle("Model 4") +
  theme(plot.title = element_text(colour="black", size=11.5),
        axis.title.x = element_text(face="plain",colour="black", size=11.5), axis.title.y = element_text(face="plain",colour="black", size=10),
        axis.text.x=element_text(size=10, color = "black", angle=45), axis.text.y=element_text(size=10, color = "black"))


# VIOLENCE - ES low
pred1 <- predict(fit_violxes,
                 newdata = data.frame(VIOLENCE="1", ES="low", STRESS="some",
                                      HEALTH_cont=0, SAD="0",
                                      GRADE_cont=0, SEX=0, RESIDENTIAL="family"),
                 type="response")
pred0 <- predict(fit_violxes,
                 newdata = data.frame(VIOLENCE="0", ES="low", STRESS="some",
                                      HEALTH_cont=0, SAD="0",
                                      GRADE_cont=0, SEX=0, RESIDENTIAL="family"),
                 type="response")
(RR_viol_es_low <-(pred1/pred0)[1])
betas <- as.vector(coef(fit_violxes))

se_viol_es_low <- deltamethod( ~(1 + exp(-x1 -0*x2 -1*x3 -0*x4 - 0*x5 -0*x6 - 0*x7 - 0*x8 - 0*x9 - 0*x10 - 0*x11 - 0*x12 -0*x13 -0*x14 -0*x15 -0*x16 - 0*x17 -0*x18 - 0*x19 -0*x20 - 0*x21)) /
                                 (1 + exp(-x1 -1*x2 -1*x3 -0*x4 - 0*x5 -0*x6 - 0*x7 - 0*x8 - 0*x9 - 0*x10 - 0*x11 - 0*x12 -0*x13 -0*x14 -0*x15 -0*x16 - 0*x17 -1*x18 - 0*x19 -0*x20 - 0*x21)), 
                               betas, vcov(fit_sadxes) 
); se_viol_es_low


#viol - ES mid-low
pred1 <- predict(fit_violxes,
                 newdata = data.frame(VIOLENCE="1", ES="mid-low", STRESS="some",
                                      HEALTH_cont=0, SAD="0",
                                      GRADE_cont=0, SEX=0, RESIDENTIAL="family"),
                 type="response")
pred0 <- predict(fit_violxes,
                 newdata = data.frame(VIOLENCE="0", ES="mid-low", STRESS="some",
                                      HEALTH_cont=0, SAD="0",
                                      GRADE_cont=0, SEX=0, RESIDENTIAL="family"),
                 type="response")
(RR_viol_es_midlow <-(pred1/pred0)[1])
betas <- as.vector(coef(fit_violxes))
se_viol_es_midlow <- deltamethod( ~(1 + exp(-x1 -0*x2 -0*x3 -1*x4 - 0*x5 -0*x6 - 0*x7 - 0*x8 - 0*x9 - 0*x10 - 0*x11 - 0*x12 -0*x13 -0*x14 -0*x15 -0*x16 - 0*x17 -0*x18 - 0*x19 -0*x20 - 0*x21)) /
                                    (1 + exp(-x1 -1*x2 -0*x3 -1*x4 - 0*x5 -0*x6 - 0*x7 - 0*x8 - 0*x9 - 0*x10 - 0*x11 - 0*x12 -0*x13 -0*x14 -0*x15 -0*x16 - 0*x17 -0*x18 - 1*x19 -0*x20 - 0*x21)), 
                                  betas, vcov(fit_violxes) 
); se_viol_es_midlow


#viol - ES mid
pred1 <- predict(fit_violxes,
                 newdata = data.frame(VIOLENCE="1", ES="mid", STRESS="some",
                                      HEALTH_cont=0, SAD="0",
                                      GRADE_cont=0, SEX=0, RESIDENTIAL="family"),
                 type="response")
pred0 <- predict(fit_violxes,
                 newdata = data.frame(VIOLENCE="0", ES="mid", STRESS="some",
                                      HEALTH_cont=0, SAD="0",
                                      GRADE_cont=0, SEX=0, RESIDENTIAL="family"),
                 type="response")
(RR_viol_es_mid <-(pred1/pred0)[1])
betas <- as.vector(coef(fit_violxes))
se_viol_es_mid <- deltamethod( ~(1 + exp(-x1 -0*x2 -0*x3 -0*x4 - 0*x5 -0*x6 - 0*x7 - 0*x8 - 0*x9 - 0*x10 - 0*x11 - 0*x12 -0*x13 -0*x14 -0*x15 -0*x16 - 0*x17 -0*x18 - 0*x19 -0*x20 - 0*x21)) /
                                 (1 + exp(-x1 -1*x2 -0*x3 -0*x4 - 0*x5 -0*x6 - 0*x7 - 0*x8 - 0*x9 - 0*x10 - 0*x11 - 0*x12 -0*x13 -0*x14 -0*x15 -0*x16 - 0*x17 -0*x18 - 0*x19 -0*x20 - 0*x21)), 
                               betas, vcov(fit_violxes) 
); se_viol_es_mid


#viol - ES mid-high
pred1 <- predict(fit_violxes,
                 newdata = data.frame(VIOLENCE="1", 
                                      ES="mid-high", STRESS="some", HEALTH_cont=0, SAD="0",
                                      GRADE_cont=0, SEX=0, RESIDENTIAL="family"),
                 type="response")
pred0 <- predict(fit_violxes,
                 newdata = data.frame(VIOLENCE="0", 
                                      ES="mid-high", STRESS="some", HEALTH_cont=0, SAD="0",
                                      GRADE_cont=0, SEX=0, RESIDENTIAL="family"),
                 type="response")
(RR_viol_es_midhigh <-(pred1/pred0)[1])
betas <- as.vector(coef(fit_violxes))
se_viol_es_midhigh <- deltamethod( ~(1 + exp(-x1 -0*x2 -0*x3 -0*x4 - 1*x5 -0*x6 - 0*x7 - 0*x8 - 0*x9 - 0*x10 - 0*x11 - 0*x12 -0*x13 -0*x14 -0*x15 -0*x16 - 0*x17 -0*x18 - 0*x19 -0*x20 - 0*x21)) /
                                     (1 + exp(-x1 -1*x2 -0*x3 -0*x4 - 1*x5 -0*x6 - 0*x7 - 0*x8 - 0*x9 - 0*x10 - 0*x11 - 0*x12 -0*x13 -0*x14 -0*x15 -0*x16 - 0*x17 -0*x18 - 0*x19 -1*x20 - 0*x21)), 
                                   betas, vcov(fit_violxes) 
); se_viol_es_midhigh


#viol - ES high
pred1 <- predict(fit_violxes,
                 newdata = data.frame(VIOLENCE="1", 
                                      ES="high", STRESS="some", HEALTH_cont=0, SAD="0",
                                      GRADE_cont=0, SEX=0, RESIDENTIAL="family"),
                 type="response")
pred0 <- predict(fit_violxes,
                 newdata = data.frame(VIOLENCE="0",
                                      ES="high", STRESS="some", HEALTH_cont=0, SAD="0",
                                      GRADE_cont=0, SEX=0, RESIDENTIAL="family"),
                 type="response")
(RR_viol_es_high <-(pred1/pred0)[1])
betas <- as.vector(coef(fit_violxes))
se_viol_es_high <- deltamethod( ~(1 + exp(-x1 -0*x2 -0*x3 -0*x4 - 0*x5 -1*x6 - 0*x7 - 0*x8 - 0*x9 - 0*x10 - 0*x11 - 0*x12 -0*x13 -0*x14 -0*x15 -0*x16 - 0*x17 -0*x18 - 0*x19 -0*x20 - 0*x21)) /
                                  (1 + exp(-x1 -1*x2 -0*x3 -0*x4 - 0*x5 -1*x6 - 0*x7 - 0*x8 - 0*x9 - 0*x10 - 0*x11 - 0*x12 -0*x13 -0*x14 -0*x15 -0*x16 - 0*x17 -0*x18 - 0*x19 -0*x20 - 1*x21)), 
                                betas, vcov(fit_violxes) 
); se_viol_es_high


df_viol_es <- data.frame(
                         ES=c("low", "mid-low", "mid", "mid-high", "high"),
                         RR=c(RR_viol_es_low, RR_viol_es_midlow, RR_viol_es_mid, RR_viol_es_midhigh, RR_viol_es_high),
                         Lower=c(RR_viol_es_low - 1.645*se_viol_es_low, RR_viol_es_midlow - 1.645*se_viol_es_midlow,
                                 RR_viol_es_mid - 1.645*se_viol_es_mid, RR_viol_es_midhigh - 1.645*se_viol_es_midhigh, RR_viol_es_high - 1.645*se_viol_es_high),
                         Upper=c(RR_viol_es_low + 1.645*se_viol_es_low, RR_viol_es_midlow + 1.645*se_viol_es_midlow,
                                 RR_viol_es_mid + 1.645*se_viol_es_mid, RR_viol_es_midhigh + 1.645*se_viol_es_midhigh, RR_viol_es_high + 1.645*se_viol_es_high)
)

df_viol_es$ES <- factor( df_viol_es$ES, levels = c("low", "mid-low", "mid", "mid-high", "high")  )

plot_viol_es <- df_viol_es %>% 
  ggplot(aes(x = ES, 
             y = RR, 
             ymin=Lower,
             ymax=Upper)) +
  geom_point(size=2.5) +
  geom_errorbar(width = 0.4, size=1) +
  labs(x = "Economic Status",
       y = "Conditional Relative Risk") +
  theme_minimal() +
  ggtitle("Model 5") +
  theme(plot.title = element_text(colour="black", size=11.5),
        axis.title.x = element_text(face="plain",colour="black", size=11.5), axis.title.y = element_text(face="plain",colour="black", size=10),
        axis.text.x=element_text(size=10, color = "black", angle=45), axis.text.y=element_text(size=10, color = "black"))
```

```
(
  RRplot <- ggarrange(plot_sad_str, plot_viol_str, plot_sad_es, plot_viol_es,
                     labels = c("a", "b", "c", "d"),
                     ncol = 4, nrow=1, 
                     font.label = list(size = 11.5))
  )
```

Plot the difference between the predicted probabilities P(exposed)-P(not exposed) (Fig 4)

```
# SAD - STRESS none
pred1 <- predict(fit_sadxstr,
                 newdata = data.frame(SAD="1", STRESS="none", ES="mid",
                                      HEALTH_cont=0, VIOLENCE="0",
                                      GRADE_cont=0, SEX=0, RESIDENTIAL="family"),
                 type="response")
pred0 <- predict(fit_sadxstr,
                 newdata = data.frame(SAD="0", STRESS="none", ES="mid",
                                      HEALTH_cont=0, VIOLENCE="0",
                                      GRADE_cont=0, SEX=0, RESIDENTIAL="family"),
                 type="response")
(Diff_sad_str_none <-(pred1-pred0)[1])
betas <- as.vector(coef(fit_sadxstr))

se_sad_str_none <- deltamethod(  ~(1/(1 + exp(-x1 -0*x2 -1*x3 -0*x4 - 0*x5 -0*x6 - 0*x7 - 0*x8 - 0*x9 - 0*x10 - 0*x11 - 0*x12 -0*x13 -0*x14 -0*x15 -0*x16 - 0*x17 -0*x18 - 0*x19 -0*x20 - 0*x21)))-
                                   (1/(1 + exp(-x1 -1*x2 -1*x3 -0*x4 - 0*x5 -0*x6 - 0*x7 - 0*x8 - 0*x9 - 0*x10 - 0*x11 - 0*x12 -0*x13 -0*x14 -0*x15 -0*x16 - 0*x17 -1*x18 - 0*x19 -0*x20 - 0*x21))),
                                betas, vcov(fit_sadxstr) 
); se_sad_str_none


#SAD - STRESS low
pred1 <- predict(fit_sadxstr,
                 newdata = data.frame(SAD="1", STRESS="low", ES="mid",
                                      HEALTH_cont=0, VIOLENCE="0",
                                      GRADE_cont=0, SEX=0, RESIDENTIAL="family"),
                 type="response")
pred0 <- predict(fit_sadxstr,
                 newdata = data.frame(SAD="0", STRESS="low", ES="mid",
                                      HEALTH_cont=0, VIOLENCE="0",
                                      GRADE_cont=0, SEX=0, RESIDENTIAL="family"),
                 type="response")
(Diff_sad_str_low <-(pred1-pred0)[1])
betas <- as.vector(coef(fit_sadxstr))
se_sad_str_low <- deltamethod( ~(1/(1 + exp(-x1 -0*x2 -0*x3 -1*x4 - 0*x5 -0*x6 - 0*x7 - 0*x8 - 0*x9 - 0*x10 - 0*x11 - 0*x12 -0*x13 -0*x14 -0*x15 -0*x16 - 0*x17 -0*x18 - 0*x19 -0*x20 - 0*x21))) -
                                   (1/(1 + exp(-x1 -1*x2 -0*x3 -1*x4 - 0*x5 -0*x6 - 0*x7 - 0*x8 - 0*x9 - 0*x10 - 0*x11 - 0*x12 -0*x13 -0*x14 -0*x15 -0*x16 - 0*x17 -0*x18 - 1*x19 -0*x20 - 0*x21))), 
                                betas, vcov(fit_sadxstr) 
); se_sad_str_low


#SAD - STRESS some
pred1 <- predict(fit_sadxstr,
                 newdata = data.frame(SAD="1", STRESS="some", ES="mid",
                                      HEALTH_cont=0, VIOLENCE="0",
                                      GRADE_cont=0, SEX=0, RESIDENTIAL="family"),
                 type="response")
pred0 <- predict(fit_sadxstr,
                 newdata = data.frame(SAD="0", STRESS="some", ES="mid",
                                      HEALTH_cont=0, VIOLENCE="0",
                                      GRADE_cont=0, SEX=0, RESIDENTIAL="family"),
                 type="response")
(Diff_sad_str_some <-(pred1-pred0)[1])
betas <- as.vector(coef(fit_sadxstr))
se_sad_str_some <- deltamethod( ~(1/(1 + exp(-x1 -0*x2 -0*x3 -0*x4 - 0*x5 -0*x6 - 0*x7 - 0*x8 - 0*x9 - 0*x10 - 0*x11 - 0*x12 -0*x13 -0*x14 -0*x15 -0*x16 - 0*x17 -0*x18 - 0*x19 -0*x20 - 0*x21))) -
                                    (1/(1 + exp(-x1 -1*x2 -0*x3 -0*x4 - 0*x5 -0*x6 - 0*x7 - 0*x8 - 0*x9 - 0*x10 - 0*x11 - 0*x12 -0*x13 -0*x14 -0*x15 -0*x16 - 0*x17 -0*x18 - 0*x19 -0*x20 - 0*x21))), 
                               betas, vcov(fit_sadxstr) 
); se_sad_str_some


#SAD - STRESS high
pred1 <- predict(fit_sadxstr,
                 newdata = data.frame(SAD="1", STRESS="high", ES="mid",
                                      HEALTH_cont=0, VIOLENCE="0",
                                      GRADE_cont=0, SEX=0, RESIDENTIAL="family"),
                 type="response")
pred0 <- predict(fit_sadxstr,
                 newdata = data.frame(SAD="0", STRESS="high", ES="mid",
                                      HEALTH_cont=0, VIOLENCE="0",
                                      GRADE_cont=0, SEX=0, RESIDENTIAL="family"),
                 type="response")
(Diff_sad_str_high <-(pred1-pred0)[1])
betas <- as.vector(coef(fit_sadxstr))
se_sad_str_high <- deltamethod( ~(1/(1 + exp(-x1 -0*x2 -0*x3 -0*x4 - 1*x5 -0*x6 - 0*x7 - 0*x8 - 0*x9 - 0*x10 - 0*x11 - 0*x12 -0*x13 -0*x14 -0*x15 -0*x16 - 0*x17 -0*x18 - 0*x19 -0*x20 - 0*x21))) -
                                    (1/(1 + exp(-x1 -1*x2 -0*x3 -0*x4 - 1*x5 -0*x6 - 0*x7 - 0*x8 - 0*x9 - 0*x10 - 0*x11 - 0*x12 -0*x13 -0*x14 -0*x15 -0*x16 - 0*x17 -0*x18 - 0*x19 -1*x20 - 0*x21))), 
                                betas, vcov(fit_sadxstr) 
); se_sad_str_high


#SAD - STRESS very high
pred1 <- predict(fit_sadxstr,
                 newdata = data.frame(SAD="1", STRESS="very high", ES="mid",
                                      HEALTH_cont=0, VIOLENCE="0",
                                      GRADE_cont=0, SEX=0, RESIDENTIAL="family"),
                 type="response")
pred0 <- predict(fit_sadxstr,
                 newdata = data.frame(SAD="0", STRESS="very high", ES="mid",
                                      HEALTH_cont=0, VIOLENCE="0",
                                      GRADE_cont=0, SEX=0, RESIDENTIAL="family"),
                 type="response")
(Diff_sad_str_vhigh <-(pred1-pred0)[1])
betas <- as.vector(coef(fit_sadxstr))
se_sad_str_vhigh <- deltamethod( ~(1/(1 + exp(-x1 -0*x2 -0*x3 -0*x4 - 0*x5 -1*x6 - 0*x7 - 0*x8 - 0*x9 - 0*x10 - 0*x11 - 0*x12 -0*x13 -0*x14 -0*x15 -0*x16 - 0*x17 -0*x18 - 0*x19 -0*x20 - 0*x21))) -
                                     (1/(1 + exp(-x1 -1*x2 -0*x3 -0*x4 - 0*x5 -1*x6 - 0*x7 - 0*x8 - 0*x9 - 0*x10 - 0*x11 - 0*x12 -0*x13 -0*x14 -0*x15 -0*x16 - 0*x17 -0*x18 - 0*x19 -0*x20 - 1*x21))), 
                                betas, vcov(fit_sadxstr) 
); se_sad_str_vhigh


df_sad_str <- data.frame(
           STRESS= c("very high", "high", "some", "low", "none"),
           Diff=c(Diff_sad_str_vhigh, Diff_sad_str_high, Diff_sad_str_some, Diff_sad_str_low, Diff_sad_str_none),
           Lower=c(Diff_sad_str_vhigh - 1.645*se_sad_str_vhigh, Diff_sad_str_high - 1.645*se_sad_str_high,
                   Diff_sad_str_some - 1.645*se_sad_str_some, Diff_sad_str_low - 1.645*se_sad_str_low, Diff_sad_str_none - 1.645*se_sad_str_none),
           Upper=c(Diff_sad_str_vhigh + 1.645*se_sad_str_vhigh, Diff_sad_str_high + 1.645*se_sad_str_high,
                   Diff_sad_str_some + 1.645*se_sad_str_some, Diff_sad_str_low + 1.645*se_sad_str_low, Diff_sad_str_none + 1.645*se_sad_str_none)
)

df_sad_str$STRESS <- factor( df_sad_str$STRESS, levels = c("very high","high","some","low","none")  )

plot_sad_str <- df_sad_str %>% 
  ggplot(aes(x = STRESS, 
             y = Diff, 
             ymin=Lower,
             ymax=Upper)) +
  geom_point(size=2.5) +
  geom_errorbar(width = 0.55, size=1) +
  #ylim(0, 0.5) +
  labs(x = "Stress",
       y = "Difference in predicted probability") +
  theme_minimal() +
  ggtitle("Model 2") +
  theme(plot.title = element_text(colour="black", size=11.5),
        axis.title.x = element_text(face="plain",colour="black", size=11.5), axis.title.y = element_text(face="plain",colour="black", size=10),
        axis.text.x=element_text(size=10, color = "black", angle=45), axis.text.y=element_text(size=10, color = "black"))
  


# VIOLENCE - STRESS none
pred1 <- predict(fit_violxstr,
                 newdata = data.frame(VIOLENCE="1", STRESS="none", ES="mid",
                                      HEALTH_cont=0, SAD="0",
                                      GRADE_cont=0, SEX=0, RESIDENTIAL="family"),
                 type="response")
pred0 <- predict(fit_violxstr,
                 newdata = data.frame(VIOLENCE="0", STRESS="none", ES="mid",
                                      HEALTH_cont=0, SAD="0",
                                      GRADE_cont=0, SEX=0, RESIDENTIAL="family"),
                 type="response")
(Diff_viol_str_none <-(pred1-pred0)[1])
betas <- as.vector(coef(fit_violxstr))
se_viol_str_none <- deltamethod( ~(1/(1 + exp(-x1 -0*x2 -1*x3 -0*x4 - 0*x5 -0*x6 - 0*x7 - 0*x8 - 0*x9 - 0*x10 - 0*x11 - 0*x12 -0*x13 -0*x14 -0*x15 -0*x16 - 0*x17 -0*x18 - 0*x19 -0*x20 - 0*x21))) -
                                     (1/(1 + exp(-x1 -1*x2 -1*x3 -0*x4 - 0*x5 -0*x6 - 0*x7 - 0*x8 - 0*x9 - 0*x10 - 0*x11 - 0*x12 -0*x13 -0*x14 -0*x15 -0*x16 - 0*x17 -1*x18 - 0*x19 -0*x20 - 0*x21))), 
                                betas, vcov(fit_sadxstr) 
); se_viol_str_none


#viol - STRESS low
pred1 <- predict(fit_violxstr,
                 newdata = data.frame(VIOLENCE="1", STRESS="low", ES="mid",
                                      HEALTH_cont=0, SAD="0",
                                      GRADE_cont=0, SEX=0, RESIDENTIAL="family"),
                 type="response")
pred0 <- predict(fit_violxstr,
                 newdata = data.frame(VIOLENCE="0", STRESS="low", ES="mid",
                                      HEALTH_cont=0, SAD="0",
                                      GRADE_cont=0, SEX=0, RESIDENTIAL="family"),
                 type="response")
(Diff_viol_str_low <-(pred1-pred0)[1])
betas <- as.vector(coef(fit_violxstr))
se_viol_str_low <- deltamethod( ~(1/(1 + exp(-x1 -0*x2 -0*x3 -1*x4 - 0*x5 -0*x6 - 0*x7 - 0*x8 - 0*x9 - 0*x10 - 0*x11 - 0*x12 -0*x13 -0*x14 -0*x15 -0*x16 - 0*x17 -0*x18 - 0*x19 -0*x20 - 0*x21))) -
                                    (1/(1 + exp(-x1 -1*x2 -0*x3 -1*x4 - 0*x5 -0*x6 - 0*x7 - 0*x8 - 0*x9 - 0*x10 - 0*x11 - 0*x12 -0*x13 -0*x14 -0*x15 -0*x16 - 0*x17 -0*x18 - 1*x19 -0*x20 - 0*x21))), 
                                betas, vcov(fit_violxstr) 
); se_viol_str_low


#viol - STRESS some
pred1 <- predict(fit_violxstr,
                 newdata = data.frame(VIOLENCE="1", STRESS="some", ES="mid",
                                      HEALTH_cont=0, SAD="0",
                                      GRADE_cont=0, SEX=0, RESIDENTIAL="family"),
                 type="response")
pred0 <- predict(fit_violxstr,
                 newdata = data.frame(VIOLENCE="0", STRESS="some", ES="mid",
                                      HEALTH_cont=0, SAD="0",
                                      GRADE_cont=0, SEX=0, RESIDENTIAL="family"),
                 type="response")
(Diff_viol_str_some <-(pred1-pred0)[1])
betas <- as.vector(coef(fit_violxstr))
se_viol_str_some <- deltamethod( ~(1/(1 + exp(-x1 -0*x2 -0*x3 -0*x4 - 0*x5 -0*x6 - 0*x7 - 0*x8 - 0*x9 - 0*x10 - 0*x11 - 0*x12 -0*x13 -0*x14 -0*x15 -0*x16 - 0*x17 -0*x18 - 0*x19 -0*x20 - 0*x21))) -
                                   (1/(1 + exp(-x1 -1*x2 -0*x3 -0*x4 - 0*x5 -0*x6 - 0*x7 - 0*x8 - 0*x9 - 0*x10 - 0*x11 - 0*x12 -0*x13 -0*x14 -0*x15 -0*x16 - 0*x17 -0*x18 - 0*x19 -0*x20 - 0*x21))), 
                                 betas, vcov(fit_violxstr) 
); se_viol_str_some


#viol - STRESS high
pred1 <- predict(fit_violxstr,
                 newdata = data.frame(VIOLENCE="1", 
                                      STRESS="high", ES="mid", HEALTH_cont=0, SAD="0",
                                      GRADE_cont=0, SEX=0, RESIDENTIAL="family"),
                 type="response")
pred0 <- predict(fit_violxstr,
                 newdata = data.frame(VIOLENCE="0", 
                                      STRESS="high", ES="mid", HEALTH_cont=0, SAD="0",
                                      GRADE_cont=0, SEX=0, RESIDENTIAL="family"),
                 type="response")
(Diff_viol_str_high <-(pred1-pred0)[1])
betas <- as.vector(coef(fit_violxstr))
se_viol_str_high <- deltamethod( ~(1/(1 + exp(-x1 -0*x2 -0*x3 -0*x4 - 1*x5 -0*x6 - 0*x7 - 0*x8 - 0*x9 - 0*x10 - 0*x11 - 0*x12 -0*x13 -0*x14 -0*x15 -0*x16 - 0*x17 -0*x18 - 0*x19 -0*x20 - 0*x21))) -
                                     (1/(1 + exp(-x1 -1*x2 -0*x3 -0*x4 - 1*x5 -0*x6 - 0*x7 - 0*x8 - 0*x9 - 0*x10 - 0*x11 - 0*x12 -0*x13 -0*x14 -0*x15 -0*x16 - 0*x17 -0*x18 - 0*x19 -1*x20 - 0*x21))), 
                                 betas, vcov(fit_violxstr) 
); se_viol_str_high


#viol - STRESS very high
pred1 <- predict(fit_violxstr,
                 newdata = data.frame(VIOLENCE="1", 
                                      STRESS="very high", ES="mid", HEALTH_cont=0, SAD="0",
                                      GRADE_cont=0, SEX=0, RESIDENTIAL="family"),
                 type="response")
pred0 <- predict(fit_violxstr,
                 newdata = data.frame(VIOLENCE="0",
                                      STRESS="very high", ES="mid", HEALTH_cont=0, SAD="0",
                                      GRADE_cont=0, SEX=0, RESIDENTIAL="family"),
                 type="response")
(Diff_viol_str_vhigh <-(pred1-pred0)[1])
betas <- as.vector(coef(fit_violxstr))
se_viol_str_vhigh <- deltamethod( ~(1/(1 + exp(-x1 -0*x2 -0*x3 -0*x4 - 0*x5 -1*x6 - 0*x7 - 0*x8 - 0*x9 - 0*x10 - 0*x11 - 0*x12 -0*x13 -0*x14 -0*x15 -0*x16 - 0*x17 -0*x18 - 0*x19 -0*x20 - 0*x21))) -
                                      (1/(1 + exp(-x1 -1*x2 -0*x3 -0*x4 - 0*x5 -1*x6 - 0*x7 - 0*x8 - 0*x9 - 0*x10 - 0*x11 - 0*x12 -0*x13 -0*x14 -0*x15 -0*x16 - 0*x17 -0*x18 - 0*x19 -0*x20 - 1*x21))), 
                                  betas, vcov(fit_violxstr) 
); se_viol_str_vhigh


df_viol_str <- data.frame(           
                          STRESS= c("very high", "high", "some", "low", "none"),
                          Diff= c(Diff_viol_str_vhigh, Diff_viol_str_high, Diff_viol_str_some, Diff_viol_str_low, Diff_viol_str_none),
                          Lower= c(Diff_viol_str_vhigh - 1.645*se_viol_str_vhigh, Diff_viol_str_high - 1.645*se_viol_str_high,
                                  Diff_viol_str_some - 1.645*se_viol_str_some, Diff_viol_str_low - 1.645*se_viol_str_low, Diff_viol_str_none - 1.645*se_viol_str_none),
                          Upper= c(Diff_viol_str_vhigh + 1.645*se_viol_str_vhigh, Diff_viol_str_high + 1.645*se_viol_str_high,
                                  Diff_viol_str_some + 1.645*se_viol_str_some, Diff_viol_str_low + 1.645*se_viol_str_low, Diff_viol_str_none + 1.645*se_viol_str_none)
)

df_viol_str$STRESS <- factor( df_viol_str$STRESS, levels = c("very high","high","some","low","none")  )

plot_viol_str <- df_viol_str %>% 
  ggplot(aes(x = STRESS, 
             y = Diff, 
             ymin=Lower,
             ymax=Upper)) +
  geom_point(size=2.5) +
  geom_errorbar(width = 0.55, size=1) +
  #ylim(0, 0.5) +
  labs(x = "Stress",
       y = "Difference in predicted probability") +
  theme_minimal() +
  ggtitle("Model 3") +
  theme(plot.title = element_text(colour="black", size=11.5),
        axis.title.x = element_text(face="plain",colour="black", size=11.5), axis.title.y = element_text(face="plain",colour="black", size=10),
        axis.text.x=element_text(size=10, color = "black", angle=45), axis.text.y=element_text(size=10, color = "black"))


# SAD - ES low

pred1 <- predict(fit_sadxes,
                 newdata = data.frame(SAD="1", ES="low", STRESS="some",
                                      HEALTH_cont=0, VIOLENCE="0",
                                      GRADE_cont=0, SEX=0, RESIDENTIAL="family"),
                 type="response")
pred0 <- predict(fit_sadxes,
                 newdata = data.frame(SAD="0", ES="low", STRESS="some",
                                      HEALTH_cont=0, VIOLENCE="0",
                                      GRADE_cont=0, SEX=0, RESIDENTIAL="family"),
                 type="response")
(Diff_sad_es_low <-(pred1-pred0)[1])
betas <- as.vector(coef(fit_sadxes))
se_sad_es_low <- deltamethod( ~(1/(1 + exp(-x1 -0*x2 -1*x3 -0*x4 - 0*x5 -0*x6 - 0*x7 - 0*x8 - 0*x9 - 0*x10 - 0*x11 - 0*x12 -0*x13 -0*x14 -0*x15 -0*x16 - 0*x17 -0*x18 - 0*x19 -0*x20 - 0*x21))) -
                                  (1/(1 + exp(-x1 -1*x2 -1*x3 -0*x4 - 0*x5 -0*x6 - 0*x7 - 0*x8 - 0*x9 - 0*x10 - 0*x11 - 0*x12 -0*x13 -0*x14 -0*x15 -0*x16 - 0*x17 -1*x18 - 0*x19 -0*x20 - 0*x21))), 
                              betas, vcov(fit_sadxes) 
); se_sad_es_low


#SAD - ES mid-low
pred1 <- predict(fit_sadxes,
                 newdata = data.frame(SAD="1", ES="mid-low", STRESS="some",
                                      HEALTH_cont=0, VIOLENCE="0",
                                      GRADE_cont=0, SEX=0, RESIDENTIAL="family"),
                 type="response")
pred0 <- predict(fit_sadxes,
                 newdata = data.frame(SAD="0", ES="mid-low", STRESS="some",
                                      HEALTH_cont=0, VIOLENCE="0",
                                      GRADE_cont=0, SEX=0, RESIDENTIAL="family"),
                 type="response")
(Diff_sad_es_midlow <-(pred1-pred0)[1])
betas <- as.vector(coef(fit_sadxes))
se_sad_es_midlow <- deltamethod( ~(1/(1 + exp(-x1 -0*x2 -0*x3 -1*x4 - 0*x5 -0*x6 - 0*x7 - 0*x8 - 0*x9 - 0*x10 - 0*x11 - 0*x12 -0*x13 -0*x14 -0*x15 -0*x16 - 0*x17 -0*x18 - 0*x19 -0*x20 - 0*x21))) -
                                     (1/(1 + exp(-x1 -1*x2 -0*x3 -1*x4 - 0*x5 -0*x6 - 0*x7 - 0*x8 - 0*x9 - 0*x10 - 0*x11 - 0*x12 -0*x13 -0*x14 -0*x15 -0*x16 - 0*x17 -0*x18 - 1*x19 -0*x20 - 0*x21))), 
                                 betas, vcov(fit_sadxes) 
); se_sad_es_midlow


#SAD - ES mid
pred1 <- predict(fit_sadxes,
                 newdata = data.frame(SAD="1", ES="mid", STRESS="some",
                                      HEALTH_cont=0, VIOLENCE="0",
                                      GRADE_cont=0, SEX=0, RESIDENTIAL="family"),
                 type="response")
pred0 <- predict(fit_sadxes,
                 newdata = data.frame(SAD="0", ES="mid", STRESS="some",
                                      HEALTH_cont=0, VIOLENCE="0",
                                      GRADE_cont=0, SEX=0, RESIDENTIAL="family"),
                 type="response")
(Diff_sad_es_mid <-(pred1-pred0)[1])
betas <- as.vector(coef(fit_sadxes))
se_sad_es_mid <- deltamethod( ~(1/(1 + exp(-x1 -0*x2 -0*x3 -0*x4 - 0*x5 -0*x6 - 0*x7 - 0*x8 - 0*x9 - 0*x10 - 0*x11 - 0*x12 -0*x13 -0*x14 -0*x15 -0*x16 - 0*x17 -0*x18 - 0*x19 -0*x20 - 0*x21))) -
                                  (1/(1 + exp(-x1 -1*x2 -0*x3 -0*x4 - 0*x5 -0*x6 - 0*x7 - 0*x8 - 0*x9 - 0*x10 - 0*x11 - 0*x12 -0*x13 -0*x14 -0*x15 -0*x16 - 0*x17 -0*x18 - 0*x19 -0*x20 - 0*x21))), 
                              betas, vcov(fit_sadxes)
); se_sad_es_mid


#SAD - ES mid-high
pred1 <- predict(fit_sadxes,
                 newdata = data.frame(SAD="1", ES="mid-high", STRESS="some",
                                      HEALTH_cont=0, VIOLENCE="0",
                                      GRADE_cont=0, SEX=0, RESIDENTIAL="family"),
                 type="response")
pred0 <- predict(fit_sadxes,
                 newdata = data.frame(SAD="0", ES="mid-high", STRESS="some",
                                      HEALTH_cont=0, VIOLENCE="0",
                                      GRADE_cont=0, SEX=0, RESIDENTIAL="family"),
                 type="response")
(Diff_sad_es_midhigh <-(pred1-pred0)[1])
betas <- as.vector(coef(fit_sadxes))
se_sad_es_midhigh <- deltamethod( ~(1/(1 + exp(-x1 -0*x2 -0*x3 -0*x4 - 1*x5 -0*x6 - 0*x7 - 0*x8 - 0*x9 - 0*x10 - 0*x11 - 0*x12 -0*x13 -0*x14 -0*x15 -0*x16 - 0*x17 -0*x18 - 0*x19 -0*x20 - 0*x21))) -
                                      (1/(1 + exp(-x1 -1*x2 -0*x3 -0*x4 - 1*x5 -0*x6 - 0*x7 - 0*x8 - 0*x9 - 0*x10 - 0*x11 - 0*x12 -0*x13 -0*x14 -0*x15 -0*x16 - 0*x17 -0*x18 - 0*x19 -1*x20 - 0*x21))), 
                                  betas, vcov(fit_sadxes) 
); se_sad_es_midhigh


#SAD - ES high
pred1 <- predict(fit_sadxes,
                 newdata = data.frame(SAD="1", ES="high", STRESS="some",
                                      HEALTH_cont=0, VIOLENCE="0",
                                      GRADE_cont=0, SEX=0, RESIDENTIAL="family"),
                 type="response")
pred0 <- predict(fit_sadxes,
                 newdata = data.frame(SAD="0", ES="high", STRESS="some",
                                      HEALTH_cont=0, VIOLENCE="0",
                                      GRADE_cont=0, SEX=0, RESIDENTIAL="family"),
                 type="response")
(Diff_sad_es_high <-(pred1-pred0)[1])
betas <- as.vector(coef(fit_sadxes))
se_sad_es_high <- deltamethod( ~(1/(1 + exp(-x1 -0*x2 -0*x3 -0*x4 - 0*x5 -1*x6 - 0*x7 - 0*x8 - 0*x9 - 0*x10 - 0*x11 - 0*x12 -0*x13 -0*x14 -0*x15 -0*x16 - 0*x17 -0*x18 - 0*x19 -0*x20 - 0*x21))) -
                                   (1/(1 + exp(-x1 -1*x2 -0*x3 -0*x4 - 0*x5 -1*x6 - 0*x7 - 0*x8 - 0*x9 - 0*x10 - 0*x11 - 0*x12 -0*x13 -0*x14 -0*x15 -0*x16 - 0*x17 -0*x18 - 0*x19 -0*x20 - 1*x21))), 
                               betas, vcov(fit_sadxes) 
); se_sad_es_high


df_sad_es <- data.frame(
                        ES=c("low", "mid-low", "mid", "mid-high", "high"),
                        Diff=c(Diff_sad_es_low, Diff_sad_es_midlow, Diff_sad_es_mid, Diff_sad_es_midhigh, Diff_sad_es_high),
                        Lower=c(Diff_sad_es_low - 1.645*se_sad_es_low, Diff_sad_es_midlow - 1.645*se_sad_es_midlow,
                                Diff_sad_es_mid - 1.645*se_sad_es_mid, Diff_sad_es_midhigh - 1.645*se_sad_es_midhigh, Diff_sad_es_high - 1.645*se_sad_es_high),
                        Upper=c(Diff_sad_es_low + 1.645*se_sad_es_low, Diff_sad_es_midlow + 1.645*se_sad_es_midlow,
                                Diff_sad_es_mid + 1.645*se_sad_es_mid, Diff_sad_es_midhigh + 1.645*se_sad_es_midhigh, Diff_sad_es_high + 1.645*se_sad_es_high)
)

df_sad_es$ES <- factor( df_sad_es$ES, levels = c("low", "mid-low", "mid", "mid-high", "high")  )

plot_sad_es <- df_sad_es %>% 
  ggplot(aes(x = ES, 
             y = Diff, 
             ymin=Lower,
             ymax=Upper)) +
  geom_point(size=2.5) +
  geom_errorbar(width = 0.55, size=1) +
  #ylim(0, 0.5) +
  labs(x = "Economic Status",
       y = "Difference in predicted probability") +
  theme_minimal() +
  ggtitle("Model 4") +
  theme(plot.title = element_text(colour="black", size=11.5),
        axis.title.x = element_text(face="plain",colour="black", size=11.5), axis.title.y = element_text(face="plain",colour="black", size=10),
        axis.text.x=element_text(size=10, color = "black", angle=45), axis.text.y=element_text(size=10, color = "black"))


# VIOLENCE - ES low
pred1 <- predict(fit_violxes,
                 newdata = data.frame(VIOLENCE="1", ES="low", STRESS="some",
                                      HEALTH_cont=0, SAD="0",
                                      GRADE_cont=0, SEX=0, RESIDENTIAL="family"),
                 type="response")
pred0 <- predict(fit_violxes,
                 newdata = data.frame(VIOLENCE="0", ES="low", STRESS="some",
                                      HEALTH_cont=0, SAD="0",
                                      GRADE_cont=0, SEX=0, RESIDENTIAL="family"),
                 type="response")
(Diff_viol_es_low <-(pred1-pred0)[1])
betas <- as.vector(coef(fit_violxes))

se_viol_es_low <- deltamethod( ~(1/(1 + exp(-x1 -0*x2 -1*x3 -0*x4 - 0*x5 -0*x6 - 0*x7 - 0*x8 - 0*x9 - 0*x10 - 0*x11 - 0*x12 -0*x13 -0*x14 -0*x15 -0*x16 - 0*x17 -0*x18 - 0*x19 -0*x20 - 0*x21))) -
                                   (1/(1 + exp(-x1 -1*x2 -1*x3 -0*x4 - 0*x5 -0*x6 - 0*x7 - 0*x8 - 0*x9 - 0*x10 - 0*x11 - 0*x12 -0*x13 -0*x14 -0*x15 -0*x16 - 0*x17 -1*x18 - 0*x19 -0*x20 - 0*x21))), 
                               betas, vcov(fit_sadxes) 
); se_viol_es_low


#viol - ES mid-low
pred1 <- predict(fit_violxes,
                 newdata = data.frame(VIOLENCE="1", ES="mid-low", STRESS="some",
                                      HEALTH_cont=0, SAD="0",
                                      GRADE_cont=0, SEX=0, RESIDENTIAL="family"),
                 type="response")
pred0 <- predict(fit_violxes,
                 newdata = data.frame(VIOLENCE="0", ES="mid-low", STRESS="some",
                                      HEALTH_cont=0, SAD="0",
                                      GRADE_cont=0, SEX=0, RESIDENTIAL="family"),
                 type="response")
(Diff_viol_es_midlow <-(pred1-pred0)[1])
betas <- as.vector(coef(fit_violxes))
se_viol_es_midlow <- deltamethod( ~(1/(1 + exp(-x1 -0*x2 -0*x3 -1*x4 - 0*x5 -0*x6 - 0*x7 - 0*x8 - 0*x9 - 0*x10 - 0*x11 - 0*x12 -0*x13 -0*x14 -0*x15 -0*x16 - 0*x17 -0*x18 - 0*x19 -0*x20 - 0*x21))) -
                                      (1/(1 + exp(-x1 -1*x2 -0*x3 -1*x4 - 0*x5 -0*x6 - 0*x7 - 0*x8 - 0*x9 - 0*x10 - 0*x11 - 0*x12 -0*x13 -0*x14 -0*x15 -0*x16 - 0*x17 -0*x18 - 1*x19 -0*x20 - 0*x21))), 
                                  betas, vcov(fit_violxes) 
); se_viol_es_midlow


#viol - ES mid
pred1 <- predict(fit_violxes,
                 newdata = data.frame(VIOLENCE="1", ES="mid", STRESS="some",
                                      HEALTH_cont=0, SAD="0",
                                      GRADE_cont=0, SEX=0, RESIDENTIAL="family"),
                 type="response")
pred0 <- predict(fit_violxes,
                 newdata = data.frame(VIOLENCE="0", ES="mid", STRESS="some",
                                      HEALTH_cont=0, SAD="0",
                                      GRADE_cont=0, SEX=0, RESIDENTIAL="family"),
                 type="response")
(Diff_viol_es_mid <-(pred1-pred0)[1])
betas <- as.vector(coef(fit_violxes))
se_viol_es_mid <- deltamethod( ~(1/(1 + exp(-x1 -0*x2 -0*x3 -0*x4 - 0*x5 -0*x6 - 0*x7 - 0*x8 - 0*x9 - 0*x10 - 0*x11 - 0*x12 -0*x13 -0*x14 -0*x15 -0*x16 - 0*x17 -0*x18 - 0*x19 -0*x20 - 0*x21))) -
                                   (1/(1 + exp(-x1 -1*x2 -0*x3 -0*x4 - 0*x5 -0*x6 - 0*x7 - 0*x8 - 0*x9 - 0*x10 - 0*x11 - 0*x12 -0*x13 -0*x14 -0*x15 -0*x16 - 0*x17 -0*x18 - 0*x19 -0*x20 - 0*x21))), 
                               betas, vcov(fit_violxes) 
); se_viol_es_mid


#viol - ES mid-high
pred1 <- predict(fit_violxes,
                 newdata = data.frame(VIOLENCE="1", 
                                      ES="mid-high", STRESS="some", HEALTH_cont=0, SAD="0",
                                      GRADE_cont=0, SEX=0, RESIDENTIAL="family"),
                 type="response")
pred0 <- predict(fit_violxes,
                 newdata = data.frame(VIOLENCE="0", 
                                      ES="mid-high", STRESS="some", HEALTH_cont=0, SAD="0",
                                      GRADE_cont=0, SEX=0, RESIDENTIAL="family"),
                 type="response")
(Diff_viol_es_midhigh <-(pred1-pred0)[1])
betas <- as.vector(coef(fit_violxes))
se_viol_es_midhigh <- deltamethod( ~(1/(1 + exp(-x1 -0*x2 -0*x3 -0*x4 - 1*x5 -0*x6 - 0*x7 - 0*x8 - 0*x9 - 0*x10 - 0*x11 - 0*x12 -0*x13 -0*x14 -0*x15 -0*x16 - 0*x17 -0*x18 - 0*x19 -0*x20 - 0*x21))) -
                                       (1/(1 + exp(-x1 -1*x2 -0*x3 -0*x4 - 1*x5 -0*x6 - 0*x7 - 0*x8 - 0*x9 - 0*x10 - 0*x11 - 0*x12 -0*x13 -0*x14 -0*x15 -0*x16 - 0*x17 -0*x18 - 0*x19 -1*x20 - 0*x21))), 
                                   betas, vcov(fit_violxes) 
); se_viol_es_midhigh


#viol - ES high
pred1 <- predict(fit_violxes,
                 newdata = data.frame(VIOLENCE="1", 
                                      ES="high", STRESS="some", HEALTH_cont=0, SAD="0",
                                      GRADE_cont=0, SEX=0, RESIDENTIAL="family"),
                 type="response")
pred0 <- predict(fit_violxes,
                 newdata = data.frame(VIOLENCE="0",
                                      ES="high", STRESS="some", HEALTH_cont=0, SAD="0",
                                      GRADE_cont=0, SEX=0, RESIDENTIAL="family"),
                 type="response")
(Diff_viol_es_high <-(pred1-pred0)[1])
betas <- as.vector(coef(fit_violxes))
se_viol_es_high <- deltamethod( ~(1/(1 + exp(-x1 -0*x2 -0*x3 -0*x4 - 0*x5 -1*x6 - 0*x7 - 0*x8 - 0*x9 - 0*x10 - 0*x11 - 0*x12 -0*x13 -0*x14 -0*x15 -0*x16 - 0*x17 -0*x18 - 0*x19 -0*x20 - 0*x21))) -
                                    (1/(1 + exp(-x1 -1*x2 -0*x3 -0*x4 - 0*x5 -1*x6 - 0*x7 - 0*x8 - 0*x9 - 0*x10 - 0*x11 - 0*x12 -0*x13 -0*x14 -0*x15 -0*x16 - 0*x17 -0*x18 - 0*x19 -0*x20 - 1*x21))), 
                                betas, vcov(fit_violxes) 
); se_viol_es_high


df_viol_es <- data.frame(
                         ES=c("low", "mid-low", "mid", "mid-high", "high"),
                         Diff=c(Diff_viol_es_low, Diff_viol_es_midlow, Diff_viol_es_mid, Diff_viol_es_midhigh, Diff_viol_es_high),
                         Lower=c(Diff_viol_es_low - 1.645*se_viol_es_low, Diff_viol_es_midlow - 1.645*se_viol_es_midlow,
                                 Diff_viol_es_mid - 1.645*se_viol_es_mid, Diff_viol_es_midhigh - 1.645*se_viol_es_midhigh, Diff_viol_es_high - 1.645*se_viol_es_high),
                         Upper=c(Diff_viol_es_low + 1.645*se_viol_es_low, Diff_viol_es_midlow + 1.645*se_viol_es_midlow,
                                 Diff_viol_es_mid + 1.645*se_viol_es_mid, Diff_viol_es_midhigh + 1.645*se_viol_es_midhigh, Diff_viol_es_high + 1.645*se_viol_es_high)
)

df_viol_es$ES <- factor( df_viol_es$ES, levels = c("low", "mid-low", "mid", "mid-high", "high")  )

plot_viol_es <- df_viol_es %>% 
  ggplot(aes(x = ES, 
             y = Diff, 
             ymin=Lower,
             ymax=Upper)) +
  geom_point(size=2.5) +
  geom_errorbar(width = 0.55, size=1) +
  #ylim(0, 0.5) +
  labs(x = "Economic Status",
       y = "Difference in predicted probability") +
  theme_minimal() +
  ggtitle("Model 5") +
  theme(plot.title = element_text(colour="black", size=11.5),
        axis.title.x = element_text(face="plain",colour="black", size=11.5), axis.title.y = element_text(face="plain",colour="black", size=10),
        axis.text.x=element_text(size=10, color = "black", angle=45), axis.text.y=element_text(size=10, color = "black"))
```

```
(
  Diffplot <- ggarrange(plot_sad_str, plot_viol_str, plot_sad_es, plot_viol_es,
                     labels = c("a", "b", "c", "d"),
                     ncol = 4, nrow=1, 
                     font.label = list(size = 11.5))
  )
```

Check VIF

```
#no interaction model
test <-glm(SUI_CON ~ SAD + STRESS1 + STRESS2 +STRESS4 +STRESS5 + ES1 + ES2 +ES4 +ES5 + VIOLENCE +
             HEALTH_cont + SEX + GRADE_cont + RESIDENTIAL, data=bigdat2s, family='binomial')
car::vif(test)
```

```
##                 GVIF Df GVIF^(1/(2*Df))
## SAD         1.073314  1        1.036009
## STRESS1     1.065722  1        1.032338
## STRESS2     1.123554  1        1.059978
## STRESS4     1.625704  1        1.275031
## STRESS5     1.653677  1        1.285954
## ES1         1.079810  1        1.039139
## ES2         1.146857  1        1.070914
## ES4         1.179317  1        1.085964
## ES5         1.136115  1        1.065887
## VIOLENCE    1.052328  1        1.025830
## HEALTH_cont 1.105015  1        1.051197
## SEX         1.049960  1        1.024676
## GRADE_cont  1.067412  1        1.033156
## RESIDENTIAL 1.071783  3        1.011621
```

```
#Stress
test <-glm(SUI_CON ~ SAD*STRESS1 + SAD*STRESS2 +SAD*STRESS4 +SAD*STRESS5 +
  HEALTH_cont + ES1 + ES2 +ES4 +ES5 + VIOLENCE + GRADE_cont + SEX + RESIDENTIAL, data=bigdat2s, family='binomial')
car::vif(test)
```

```
##                 GVIF Df GVIF^(1/(2*Df))
## SAD         3.974333  1        1.993573
## STRESS1     2.536566  1        1.592660
## STRESS2     2.378200  1        1.542141
## STRESS4     4.047308  1        2.011792
## STRESS5     4.855604  1        2.203544
## HEALTH_cont 1.101933  1        1.049730
## ES1         1.080032  1        1.039246
## ES2         1.147518  1        1.071223
## ES4         1.179251  1        1.085933
## ES5         1.135082  1        1.065402
## VIOLENCE    1.059735  1        1.029434
## GRADE_cont  1.065074  1        1.032024
## SEX         1.047563  1        1.023505
## RESIDENTIAL 1.071451  3        1.011569
## SAD:STRESS1 2.556150  1        1.598796
## SAD:STRESS2 2.389738  1        1.545878
## SAD:STRESS4 5.584980  1        2.363256
## SAD:STRESS5 5.883735  1        2.425641
```

```
test <-glm(SUI_CON ~ VIOLENCE*STRESS1 + VIOLENCE*STRESS2 +VIOLENCE*STRESS4 +VIOLENCE*STRESS5 +
             HEALTH_cont + ES1 + ES2 +ES4 +ES5 + SAD + GRADE_cont + SEX + RESIDENTIAL, data=bigdat2s, family='binomial')
car::vif(test)
```

```
##                      GVIF Df GVIF^(1/(2*Df))
## VIOLENCE         3.890426  1        1.972416
## STRESS1          1.538958  1        1.240547
## STRESS2          1.264829  1        1.124646
## STRESS4          1.706395  1        1.306290
## STRESS5          1.753253  1        1.324104
## HEALTH_cont      1.103456  1        1.050455
## ES1              1.081268  1        1.039840
## ES2              1.147040  1        1.070999
## ES4              1.179431  1        1.086016
## ES5              1.136947  1        1.066277
## SAD              1.073945  1        1.036313
## GRADE_cont       1.067679  1        1.033285
## SEX              1.051077  1        1.025221
## RESIDENTIAL      1.083853  3        1.013511
## VIOLENCE:STRESS1 1.811680  1        1.345986
## VIOLENCE:STRESS2 1.497803  1        1.223848
## VIOLENCE:STRESS4 2.170625  1        1.473304
## VIOLENCE:STRESS5 2.177046  1        1.475482
```

```
#Economic status
test <-glm(SUI_CON ~ SAD*ES1 + SAD*ES2 +SAD*ES4 +SAD*ES5 +
             STRESS1 + STRESS2 +STRESS4 +STRESS5 + HEALTH_cont + VIOLENCE + SEX + GRADE_cont + RESIDENTIAL,
           data=bigdat2s, family='binomial')
car::vif(test)
```

```
##                 GVIF Df GVIF^(1/(2*Df))
## SAD         2.288563  1        1.512800
## ES1         3.498311  1        1.870377
## ES2         3.148468  1        1.774392
## ES4         3.266793  1        1.807427
## ES5         3.150275  1        1.774901
## STRESS1     1.067459  1        1.033179
## STRESS2     1.124225  1        1.060295
## STRESS4     1.626876  1        1.275490
## STRESS5     1.654695  1        1.286350
## HEALTH_cont 1.104832  1        1.051110
## VIOLENCE    1.052496  1        1.025912
## SEX         1.049772  1        1.024584
## GRADE_cont  1.066792  1        1.032856
## RESIDENTIAL 1.072304  3        1.011703
## SAD:ES1     3.529535  1        1.878706
## SAD:ES2     3.356252  1        1.832008
## SAD:ES4     3.706969  1        1.925349
## SAD:ES5     3.244660  1        1.801294
```

```
test <-glm(SUI_CON ~ VIOLENCE*ES1 + VIOLENCE*ES2 +VIOLENCE*ES4 +VIOLENCE*ES5 +
             STRESS1 + STRESS2 +STRESS4 +STRESS5 + HEALTH_cont + SAD + SEX + GRADE_cont + RESIDENTIAL,
           data=bigdat2s, family='binomial')
car::vif(test)
```

```
##                  GVIF Df GVIF^(1/(2*Df))
## VIOLENCE     2.978022  1        1.725695
## ES1          1.188378  1        1.090127
## ES2          1.192406  1        1.091974
## ES4          1.223617  1        1.106172
## ES5          1.229757  1        1.108944
## STRESS1      1.069384  1        1.034110
## STRESS2      1.123671  1        1.060033
## STRESS4      1.625715  1        1.275035
## STRESS5      1.653860  1        1.286025
## HEALTH_cont  1.104920  1        1.051152
## SAD          1.073451  1        1.036075
## SEX          1.050158  1        1.024772
## GRADE_cont   1.068290  1        1.033581
## RESIDENTIAL  1.099328  3        1.015908
## VIOLENCE:ES1 1.449094  1        1.203783
## VIOLENCE:ES2 1.461876  1        1.209081
## VIOLENCE:ES4 1.729156  1        1.314974
## VIOLENCE:ES5 1.714796  1        1.309502
```

Try models that interact crisis with both stress and economic status, and plot.

```
#model 6
fit_sad_both_interactions <- svyglm(SUI_CON ~ SAD*STRESS + SAD*ES + HEALTH_cont + VIOLENCE + GRADE_cont + SEX + RESIDENTIAL,
                                    design=design,family=binomial("logit"))
#model 7
fit_viol_both_interactions <- svyglm(SUI_CON ~ VIOLENCE*STRESS + VIOLENCE*ES + HEALTH_cont + SAD + GRADE_cont + SEX + RESIDENTIAL,
                                     design=design,family=binomial("logit"))


#sadxstress
newdat.s1 <- data.frame(
  "SAD"= c( rep("0",5),  rep("1",5)   ),
  "STRESS"= c(rep(  c("very high","high", "some", "low", "none"),2) ),
  "HEALTH_cont"= c(rep(0,10) ), 
  "ES"= c(rep("mid",10) ),
  "VIOLENCE" = c( rep("0",10) ),
  "SEX" = c( rep(0,10) ),
  "GRADE_cont" = c(rep(0, 10)),
  "RESIDENTIAL" = c(rep("family", 10))
  )
newdat.s1

pred <- predict(fit_sad_both_interactions, newdata = newdat.s1, type="link")
df <- data.frame( "DESPAIR" = c(rep("0",5), rep("1",5)),
                  "STRESS" = c( "very high","high","some","low","none", "very high","high","some","low","none")
)
dats1 <- cbind(df, pred)
dats1$STRESS <- factor( dats1$STRESS, levels = c("very high","high","some","low","none")  )
dats1 #the 'link' column shows the logit values, the SE column the logit SEs

graphs1 <- dats1 %>% 
  ggplot(aes(x = STRESS, 
             y = logistic(link), 
             color = DESPAIR,
             ymin=logistic(link - 1.645*SE),
             ymax=logistic(link + 1.645*SE))) +
  geom_point(size=2.5) +
  geom_errorbar(width = 0.55, size=1) +
  ylim(0, 0.5) +
  labs(x = "Stress",
       color  = "Despair/Grief",
       y = "Probability of Suicidal Ideation") +
  theme_minimal() +
  ggtitle("Model 6") +
  theme(plot.title = element_text(colour="black", size=11.5),
        legend.text = element_text(colour="black",size=10), legend.title = element_text(face="plain",colour="black", size=10),legend.position="bottom",
        axis.title.x = element_text(face="plain",colour="black", size=11.5), axis.title.y = element_text(face="plain",colour="black", size=10),
        axis.text.x=element_text(size=10, color = "black", angle=45), axis.text.y=element_text(size=10, color = "black")) +
  scale_color_manual(labels = c("0"= "No", "1"=  "Yes"), values=c("blue","red"))


#violxstress
newdat.v1 <- data.frame(
  "VIOLENCE"= c( rep("0",5),  rep("1",5)   ),
  "STRESS"= c(rep(  c("very high","high", "some", "low", "none"),2) ),
  "HEALTH_cont"= c(rep(0,10) ), 
  "ES"= c(rep("mid",10) ),
  "SAD" = c( rep("0",10) ),
  "SEX" = c( rep(0,10) ),
  "GRADE_cont" = c(rep(0, 10)),
  "RESIDENTIAL" = c(rep("family", 10))
  )
newdat.v1

pred <- predict(fit_viol_both_interactions, newdata = newdat.v1, type="link")
df <- data.frame( "VIOLENCE" = c(rep("0",5), rep("1",5)),
                  "STRESS" = c( "very high","high","some","low","none", "very high","high","some","low","none")
)
datv1 <- cbind(df, pred)
datv1$STRESS <- factor( datv1$STRESS, levels = c("very high","high","some","low","none")  )
datv1 

graphv1 <- datv1 %>% 
  ggplot(aes(x = STRESS, 
             y = logistic(link), 
             color = VIOLENCE,
             ymin=logistic(link - 1.645*SE),
             ymax=logistic(link + 1.645*SE))) +
  geom_point(size=2.5) +
  geom_errorbar(width = 0.55, size=1) +
  ylim(0, 0.25) +
  labs(x = "Stress",
       color  = "Violence",
       y = "Probability of Suicidal Ideation") +
  theme_minimal() +
  ggtitle("Model 7") +
  theme(plot.title = element_text(colour="black", size=11.5),
        legend.text = element_text(colour="black",size=10), legend.title = element_text(face="plain",colour="black", size=10),legend.position="bottom",
        axis.title.x = element_text(face="plain",colour="black", size=11.5), axis.title.y = element_text(face="plain",colour="black", size=10),
        axis.text.x=element_text(size=10, color = "black", angle=45), axis.text.y=element_text(size=10, color = "black")) +
  scale_color_manual(labels = c("0"= "No", "1"=  "Yes"), values=c("blue","red"))


#sadxES
newdat.s2 <- data.frame(
  "SAD"= c( rep("0",5),  rep("1",5)   ),
  "ES"= c( rep(  c("low","mid-low", "mid", "mid-high", "high"),2) ),
  "STRESS"= c(rep("some",10) ), 
  "HEALTH_cont"= c(rep(0,10) ),
  "VIOLENCE" = c( rep("0",10) ),
  "SEX" = c( rep(0,10) ),
  "GRADE_cont" = c(rep(0, 10)),
  "RESIDENTIAL" = c(rep("family", 10))
  )

pred <- predict(fit_sad_both_interactions, newdata = newdat.s2, type="link")
df <- data.frame( "DESPAIR" = c(rep("0",5), rep("1",5)),
                  "ES" = c( "low","mid-low", "mid", "mid-high", "high", "low","mid-low", "mid", "mid-high", "high")
)
dats2 <- cbind(df, pred)
dats2$ES <- factor( dats2$ES, levels = c("low","mid-low", "mid", "mid-high", "high")  )


graphs2 <- dats2 %>% 
  ggplot(aes(x = ES, 
             y = logistic(link), 
             color = DESPAIR,
             ymin=logistic(link - 1.645*SE),
             ymax=logistic(link + 1.645*SE))) +
  geom_point(size=2.5) +
  geom_errorbar(width = 0.55, size=1) +
  ylim(0, 0.30) +
  labs(x = "Economic Status",
       color  = "Despair/Grief",
       y = "Probability of Suicidal Ideation") +
  theme_minimal() +
  ggtitle("Model 6") +
  theme(plot.title = element_text(colour="black", size=11.5),
        legend.text = element_text(colour="black",size=10), legend.title = element_text(face="plain",colour="black", size=10),legend.position="bottom",
        axis.title.x = element_text(face="plain",colour="black", size=11.5), axis.title.y = element_text(face="plain",colour="black", size=10),
        axis.text.x=element_text(size=10, color = "black", angle=45), axis.text.y=element_text(size=10, color = "black")) +
  scale_color_manual(labels = c("0"= "No", "1"=  "Yes"), values=c("#00AFBB","#FC4E07"))


#violxES
newdat.v2 <- data.frame(
  "VIOLENCE"= c( rep("0",5),  rep("1",5)   ),
  "ES"= c( rep(  c("low","mid-low", "mid", "mid-high", "high"),2) ),
  "STRESS"= c(rep("some",10) ), 
  "HEALTH_cont"= c(rep(0,10) ), 
  "SAD" = c( rep("0",10) ),
  "SEX" = c( rep(0,10) ),
  "GRADE_cont" = c(rep(0, 10)),
  "RESIDENTIAL" = c(rep("family", 10))
  )
newdat.v2

pred <- predict(fit_viol_both_interactions, newdata = newdat.v2, type="link")
df <- data.frame( "VIOLENCE" = c(rep("0",5), rep("1",5)),
                  "ES" = c( "low","mid-low", "mid", "mid-high", "high", "low","mid-low", "mid", "mid-high", "high")
)
datv2 <- cbind(df, pred)
datv2$ES <- factor( datv2$ES, levels = c("low","mid-low", "mid", "mid-high", "high")  )
datv2 

graphv2 <- datv2 %>% 
  ggplot(aes(x = ES, 
             y = logistic(link), 
             color = VIOLENCE,
             ymin=logistic(link - 1.645*SE),
             ymax=logistic(link + 1.645*SE))) +
  geom_point(size=2.5) +
  geom_errorbar(width = 0.55, size=1) +
  ylim(0, 0.2) +
  labs(x = "Economic Status",
       color  = "Violence",
       y = "Probability of Suicidal Ideation") +
  theme_minimal() +
  ggtitle("Model 7") +
  theme(plot.title = element_text(colour="black", size=11.5),
        legend.text = element_text(colour="black",size=10), legend.title = element_text(face="plain",colour="black", size=10),legend.position="bottom",
        axis.title.x = element_text(face="plain",colour="black", size=11.5), axis.title.y = element_text(face="plain",colour="black", size=10),
        axis.text.x=element_text(size=10, color = "black", angle=45), axis.text.y=element_text(size=10, color = "black")) +
  scale_color_manual(labels = c("0"= "No", "1"=  "Yes"), values=c("#00AFBB","#FC4E07"))


g1 <- ggarrange(graphs1, graphv1,
          labels = c("a", "b"),
          ncol = 2, nrow=1, font.label = list(size = 11.5), legend="top")

g2 <- ggarrange(graphs2, graphv2,
                labels = c("c", "d"),
                ncol = 2, nrow=1, font.label = list(size = 11.5), legend="top")
```

```
(g3 <-ggarrange(g1, g2, ncol=2, nrow=1))
```

### Multilevel Models with brms

#### Estimate the cluster means and compute cluster-mean-centered variables to be used in the multilevel models

```
#SAD
logit_S <- brm(SAD_numeric ~ (1|SCHOOL), data=bigdat2s, family=bernoulli(link = "logit"), 
                prior=c(
                  set_prior("normal(-1, 0.5)", class="Intercept"),
                  set_prior("cauchy(0, 1)", class="sd")
                ),
                cores=4, chains=4, iter=3000, warmup=1000, control=list(adapt_delta=.99))
get_prior(SAD_numeric ~ (1|SCHOOL), data=bigdat2s, family=bernoulli(link = "logit"))

#VIOLENCE
logit_V <- brm(VIOLENCE_numeric ~ (1|SCHOOL), data=bigdat2s, family=bernoulli(link = "logit"), 
               prior=c(
                 set_prior("normal(-3.5, 0.5)", class="Intercept"),
                 set_prior("cauchy(0, 1)", class="sd")
               ),
               cores=4, chains=4, iter=3000, warmup=1000, control=list(adapt_delta=.99))

#Stress
Mean_STRESS <- brm(STRESS_cont ~ (1|SCHOOL), data=bigdat2s, 
               prior=c(
                 set_prior("normal(2, 0.5)", class="Intercept"),
                 set_prior("cauchy(0, 5)", class="sigma"),
                 set_prior("cauchy(0, 5)", class="sd")
               ),
               cores=4, chains=4, iter=3000, warmup=1000, control=list(adapt_delta=.99))

#Economic Status
Mean_ES <- brm(STRESS_cont ~ (1|SCHOOL), data=bigdat2s, 
                   prior=c(
                     set_prior("normal(2, 0.5)", class="Intercept"),
                     set_prior("cauchy(0, 5)", class="sigma"),
                     set_prior("cauchy(0, 5)", class="sd")
                   ),
                   cores=4, chains=4, iter=3000, warmup=1000, control=list(adapt_delta=.99))
```

```
#SAD
##load logit_S.Rdata
ran <- ranef(logit_S)$SCHOOL[,1,1]
sadmean_logit <- fixef(logit_S)[1] + ran
sadmean_prob <- as.data.frame(logistic(sadmean_logit))
df <- setDT(sadmean_prob, keep.rownames = "SCHOOL")[]
bigdat2s <- merge(x=bigdat2s, y=df, by="SCHOOL", all=T)
colnames(bigdat2s)[which(names(bigdat2s) == "logistic(sadmean_logit)")] <- "SAD_smean"

bigdat2s$SADcwc <- bigdat2s$SAD_numeric - bigdat2s$SAD_smean #create a cluster-mean centered variable

#VIOLENCE
##load logit_V.Rdata
ran <- ranef(logit_V)$SCHOOL[,1,1]
violmean_logit <- fixef(logit_V)[1] + ran
violmean_prob <- as.data.frame(logistic(violmean_logit))
df <- setDT(violmean_prob, keep.rownames = "SCHOOL")[]
bigdat2s <- merge(x=bigdat2s, y=df, by="SCHOOL", all=T)
colnames(bigdat2s)[which(names(bigdat2s) == "logistic(violmean_logit)")] <- "VIOLENCE_smean"

bigdat2s$VIOLENCEcwc <- bigdat2s$VIOLENCE_numeric - bigdat2s$VIOLENCE_smean #create a cluster-mean centered variable

#Stress
##load Mean_STRESS.Rdata
ran <- ranef(Mean_STRESS)$SCHOOL[,1,1] #group-specific random effect.
sd <- VarCorr(Mean_STRESS)$SCHOOL$sd[1] #the standard deviation of the random effect.
stressmean <- as.data.frame(ran/sd) #standardize so that the sd becomes 1.
df <- setDT(stressmean, keep.rownames = "SCHOOL")[]
bigdat2s <- merge(x=bigdat2s, y=df, by="SCHOOL", all=T)
colnames(bigdat2s)[which(names(bigdat2s) == "ran/sd")] <- "STRESS_smean"


#Economic Status
##load Mean_ES.Rdata
ran <- ranef(Mean_ES)$SCHOOL[,1,1]
sd <- VarCorr(Mean_ES)$SCHOOL$sd[1]
esmean <- as.data.frame(ran/sd)
df <- setDT(esmean, keep.rownames = "SCHOOL")[]
bigdat2s <- merge(x=bigdat2s, y=df, by="SCHOOL", all=T)
colnames(bigdat2s)[which(names(bigdat2s) == "ran/sd")] <- "ES_smean"
```

```
#plot the distribution of group-means
par(mfrow=c(1,4))
temp <- bigdat2s[!duplicated(bigdat2s$SCHOOL),]
hist(temp$SAD_smean, xlab='', main="School Mean Despair", cex.lab=1.3, cex.axis=1.3, cex.main=1.3, cex.sub=1.3)
hist(temp$VIOLENCE_smean, xlab='', main="School Mean Violence", cex.lab=1.3, cex.axis=1.3, cex.main=1.3, cex.sub=1.3)
hist(temp$STRESS_smean, xlab='', main="School Mean Stress (Z-score)", cex.lab=1.3, cex.axis=1.3, cex.main=1.3, cex.sub=1.3)
hist(temp$ES_smean, xlab='', main="School Mean ES (Z-score)", cex.lab=1.3, cex.axis=1.3, cex.main=1.3, cex.sub=1.3)
```

Run multilevel models with the brms package. It takes many hours to fit each model.

```
#STRESS
brm_sadxstr1 <- brm(SUI_CON ~ SADcwc*STRESS  + SADcwc*STRESS_smean + ES + HEALTH_cont + VIOLENCE + 
                                     GRADE_cont + SEX + RESIDENTIAL + 
                             (1+ SADcwc | SCHOOL), 
                           data=bigdat2s, family='bernoulli', 
                           prior=c(
                             set_prior("normal(-3.5, 1)", class="Intercept"),
                             set_prior("normal(0, 1)", class="b"),
                             set_prior("normal(1.5, 1)", class="b", coef= "SADcwc"),
                             set_prior("cauchy(0, 1)", class="sd"),
                             set_prior("lkj(2)",class="cor")
                           ),
                           chains = 4, cores=4, iter=3000, warmup=1000, control=list(adapt_delta=.99))
```

```
summary(brm_sadxstr1)
```

```
##  Family: bernoulli 
##   Links: mu = logit 
## Formula: SUI_CON ~ SADcwc * STRESS + SADcwc * STRESS_smean + ES + HEALTH_cont + VIOLENCE + GRADE_cont + SEX + RESIDENTIAL + (1 + SADcwc | SCHOOL) 
##    Data: bigdat2s (Number of observations: 179619) 
## Samples: 4 chains, each with iter = 3000; warmup = 1000; thin = 1;
##          total post-warmup samples = 8000
## 
## Group-Level Effects: 
## ~SCHOOL (Number of levels: 2399) 
##                       Estimate Est.Error l-95% CI u-95% CI Rhat Bulk_ESS Tail_ESS
## sd(Intercept)             0.26      0.02     0.22     0.30 1.00     1593     3973
## sd(SADcwc)                0.14      0.07     0.01     0.26 1.01      480     1196
## cor(Intercept,SADcwc)    -0.38      0.26    -0.79     0.30 1.00     2820     1788
## 
## Population-Level Effects: 
##                             Estimate Est.Error l-95% CI u-95% CI Rhat Bulk_ESS Tail_ESS
## Intercept                      -3.29      0.03    -3.34    -3.24 1.00     6939     6335
## SADcwc                          2.24      0.03     2.17     2.31 1.00     5380     6196
## STRESSnone                     -0.36      0.09    -0.55    -0.18 1.00    10849     6134
## STRESSlow                      -0.71      0.05    -0.81    -0.60 1.00    10977     6494
## STRESShigh                      1.06      0.03     1.01     1.11 1.00     8477     6273
## STRESSveryhigh                  1.81      0.03     1.75     1.87 1.00     8020     6181
## STRESS_smean                    0.09      0.02     0.06     0.12 1.00     9318     6984
## ESlow                           0.46      0.04     0.37     0.54 1.00    17598     6182
## ESmidMlow                       0.31      0.02     0.27     0.36 1.00    15121     6141
## ESmidMhigh                      0.00      0.02    -0.04     0.04 1.00    16726     6518
## EShigh                          0.04      0.03    -0.02     0.10 1.00    16591     6122
## HEALTH_cont                    -0.24      0.01    -0.25    -0.22 1.00    19267     5279
## VIOLENCE1                       1.03      0.04     0.95     1.11 1.00    19742     5454
## GRADE_cont                     -0.10      0.01    -0.12    -0.09 1.00    12951     6399
## SEX                             0.25      0.02     0.21     0.28 1.00    15100     6382
## RESIDENTIALrelative             0.45      0.08     0.30     0.61 1.00    19077     5917
## RESIDENTIALdormDstudio          0.09      0.04     0.01     0.17 1.00    18824     6236
## RESIDENTIALnursuryDorphange     0.22      0.14    -0.05     0.48 1.00    22074     6432
## SADcwc:STRESSnone               1.36      0.16     1.04     1.68 1.00    10489     6575
## SADcwc:STRESSlow                0.82      0.10     0.63     1.01 1.00    10619     6403
## SADcwc:STRESShigh              -0.52      0.04    -0.60    -0.44 1.00     6430     6461
## SADcwc:STRESSveryhigh          -0.53      0.05    -0.63    -0.43 1.00     6659     6520
## SADcwc:STRESS_smean            -0.06      0.02    -0.11    -0.02 1.00    12392     6696
## 
## Samples were drawn using sampling(NUTS). For each parameter, Bulk_ESS
## and Tail_ESS are effective sample size measures, and Rhat is the potential
## scale reduction factor on split chains (at convergence, Rhat = 1).
```

```
brm_violxstr1 <- brm(SUI_CON ~ VIOLENCEcwc*STRESS  + VIOLENCEcwc*STRESS_smean + ES + HEALTH_cont + SAD + 
                     GRADE_cont + SEX + RESIDENTIAL + 
                     (1+ VIOLENCEcwc | SCHOOL), 
                   data=bigdat2s, family='bernoulli', 
                   prior=c(
                     set_prior("normal(-3.5, 1)", class="Intercept"),
                     set_prior("normal(0, 1)", class="b"),
                     set_prior("normal(1.5, 1)", class="b", coef= "SAD1"),
                     set_prior("cauchy(0, 1)", class="sd"),
                     set_prior("lkj(2)",class="cor")
                   ),
                   chains = 4, cores=4, iter=3000, warmup=1000, control=list(adapt_delta=.99))
```

```
summary(brm_violxstr1)
```

```
##  Family: bernoulli 
##   Links: mu = logit 
## Formula: SUI_CON ~ VIOLENCEcwc * STRESS + VIOLENCEcwc * STRESS_smean + ES + HEALTH_cont + SAD + GRADE_cont + SEX + RESIDENTIAL + (1 + VIOLENCEcwc | SCHOOL) 
##    Data: bigdat2s (Number of observations: 179619) 
## Samples: 4 chains, each with iter = 3000; warmup = 1000; thin = 1;
##          total post-warmup samples = 8000
## 
## Group-Level Effects: 
## ~SCHOOL (Number of levels: 2399) 
##                            Estimate Est.Error l-95% CI u-95% CI Rhat Bulk_ESS Tail_ESS
## sd(Intercept)                  0.23      0.01     0.20     0.26 1.00     3149     5048
## sd(VIOLENCEcwc)                0.17      0.12     0.01     0.41 1.00     1124     3014
## cor(Intercept,VIOLENCEcwc)     0.01      0.36    -0.69     0.72 1.00     7247     5456
## 
## Population-Level Effects: 
##                             Estimate Est.Error l-95% CI u-95% CI Rhat Bulk_ESS Tail_ESS
## Intercept                      -3.68      0.02    -3.73    -3.64 1.00    11050     6284
## VIOLENCEcwc                     1.38      0.08     1.23     1.52 1.00     5926     5785
## STRESSnone                     -0.45      0.09    -0.64    -0.28 1.00    12964     5623
## STRESSlow                      -0.71      0.05    -0.81    -0.62 1.00    14281     6227
## STRESShigh                      0.91      0.02     0.87     0.95 1.00    10656     6786
## STRESSveryhigh                  1.64      0.02     1.60     1.69 1.00     9453     6518
## STRESS_smean                   -0.01      0.01    -0.03     0.02 1.00    10965     6809
## ESlow                           0.47      0.04     0.38     0.55 1.00    17354     5798
## ESmidMlow                       0.32      0.02     0.27     0.37 1.00    14981     5512
## ESmidMhigh                      0.00      0.02    -0.04     0.04 1.00    13574     6437
## EShigh                          0.04      0.03    -0.02     0.10 1.00    14853     5861
## HEALTH_cont                    -0.24      0.01    -0.25    -0.22 1.00    17883     6171
## SAD1                            1.89      0.02     1.86     1.93 1.00    14692     5626
## GRADE_cont                     -0.10      0.01    -0.12    -0.09 1.00    11706     7092
## SEX                             0.24      0.02     0.20     0.27 1.00    13694     6914
## RESIDENTIALrelative             0.42      0.08     0.26     0.57 1.00    15309     5535
## RESIDENTIALdormDstudio          0.09      0.04     0.01     0.16 1.00    16130     5947
## RESIDENTIALnursuryDorphange     0.23      0.13    -0.03     0.49 1.00    15573     5669
## VIOLENCEcwc:STRESSnone          1.04      0.18     0.69     1.40 1.00     9440     5651
## VIOLENCEcwc:STRESSlow           0.98      0.15     0.68     1.27 1.00     9958     6919
## VIOLENCEcwc:STRESShigh         -0.57      0.10    -0.78    -0.37 1.00     7367     7066
## VIOLENCEcwc:STRESSveryhigh     -0.73      0.10    -0.93    -0.53 1.00     7366     6628
## VIOLENCEcwc:STRESS_smean        0.00      0.05    -0.09     0.10 1.00    15365     6543
## 
## Samples were drawn using sampling(NUTS). For each parameter, Bulk_ESS
## and Tail_ESS are effective sample size measures, and Rhat is the potential
## scale reduction factor on split chains (at convergence, Rhat = 1).
```

```
#ES
brm_sadxes1 <- brm(SUI_CON ~ SADcwc*ES  + SADcwc*ES_smean + STRESS + HEALTH_cont + VIOLENCE + 
                     GRADE_cont + SEX + RESIDENTIAL + 
                     (1+ SADcwc | SCHOOL), 
                   data=bigdat2s, family='bernoulli', 
                   prior=c(
                     set_prior("normal(-3.5, 1)", class="Intercept"),
                     set_prior("normal(0, 1)", class="b"),
                     set_prior("normal(1.5, 1)", class="b", coef= "SADcwc"),
                     set_prior("cauchy(0, 1)", class="sd"),
                     set_prior("lkj(2)",class="cor")
                   ),
                   chains = 4, cores=4, iter=3000, warmup=1000, control=list(adapt_delta=.99))
```

```
summary(brm_sadxes1)
```

```
##  Family: bernoulli 
##   Links: mu = logit 
## Formula: SUI_CON ~ SADcwc * ES + SADcwc * ES_smean + STRESS + HEALTH_cont + VIOLENCE + GRADE_cont + SEX + RESIDENTIAL + (1 + SADcwc | SCHOOL) 
##    Data: bigdat2s (Number of observations: 179619) 
## Samples: 4 chains, each with iter = 3000; warmup = 1000; thin = 1;
##          total post-warmup samples = 8000
## 
## Group-Level Effects: 
## ~SCHOOL (Number of levels: 2399) 
##                       Estimate Est.Error l-95% CI u-95% CI Rhat Bulk_ESS Tail_ESS
## sd(Intercept)             0.25      0.02     0.22     0.29 1.00     2435     4310
## sd(SADcwc)                0.12      0.07     0.01     0.25 1.00      349     1248
## cor(Intercept,SADcwc)    -0.16      0.30    -0.68     0.57 1.00     2321     2338
## 
## Population-Level Effects: 
##                             Estimate Est.Error l-95% CI u-95% CI Rhat Bulk_ESS Tail_ESS
## Intercept                      -3.21      0.02    -3.26    -3.17 1.00     6721     6193
## SADcwc                          1.92      0.03     1.87     1.97 1.00     5142     5995
## ESlow                           0.43      0.06     0.31     0.55 1.00     7365     6305
## ESmidMlow                       0.38      0.03     0.32     0.44 1.00     7775     6187
## ESmidMhigh                     -0.02      0.03    -0.07     0.03 1.00     7110     6256
## EShigh                         -0.03      0.04    -0.10     0.05 1.00     8343     6628
## ES_smean                        0.11      0.02     0.08     0.14 1.00     8148     6298
## STRESSnone                     -0.12      0.07    -0.26     0.02 1.00    13372     6198
## STRESSlow                      -0.61      0.05    -0.70    -0.52 1.00    11451     5792
## STRESShigh                      0.90      0.02     0.85     0.94 1.00     8592     6307
## STRESSveryhigh                  1.62      0.02     1.57     1.66 1.00     8039     6572
## HEALTH_cont                    -0.24      0.01    -0.26    -0.23 1.00    14381     6123
## VIOLENCE1                       1.11      0.04     1.02     1.19 1.00    16745     5547
## GRADE_cont                     -0.10      0.01    -0.12    -0.09 1.00    11266     6755
## SEX                             0.25      0.02     0.21     0.29 1.00    10025     5275
## RESIDENTIALrelative             0.50      0.08     0.34     0.65 1.00    17896     5072
## RESIDENTIALdormDstudio          0.10      0.04     0.02     0.18 1.00    12398     6383
## RESIDENTIALnursuryDorphange     0.26      0.14    -0.00     0.53 1.00    13940     4866
## SADcwc:ESlow                    0.10      0.10    -0.09     0.29 1.00     7484     6390
## SADcwc:ESmidMlow               -0.16      0.05    -0.26    -0.06 1.00     6843     6449
## SADcwc:ESmidMhigh               0.07      0.04    -0.02     0.15 1.00     5840     6156
## SADcwc:EShigh                   0.18      0.06     0.06     0.30 1.00     7373     6285
## SADcwc:ES_smean                -0.12      0.02    -0.16    -0.08 1.00    10748     6148
## 
## Samples were drawn using sampling(NUTS). For each parameter, Bulk_ESS
## and Tail_ESS are effective sample size measures, and Rhat is the potential
## scale reduction factor on split chains (at convergence, Rhat = 1).
```

```
brm_violxes1 <- brm(SUI_CON ~ VIOLENCEcwc*ES  + VIOLENCEcwc*ES_smean + STRESS + HEALTH_cont + SAD + 
                      GRADE_cont + SEX + RESIDENTIAL + 
                      (1+ VIOLENCEcwc | SCHOOL), 
                    data=bigdat2s, family='bernoulli', 
                    prior=c(
                      set_prior("normal(-3.5, 1)", class="Intercept"),
                      set_prior("normal(0, 1)", class="b"),
                      set_prior("normal(1.5, 1)", class="b", coef= "SAD1"),
                      set_prior("cauchy(0, 1)", class="sd"),
                      set_prior("lkj(2)",class="cor")
                    ),
                    chains = 4, cores=4, iter=3000, warmup=1000, control=list(adapt_delta=.99))
```

```
summary(brm_violxes1)
```

```
##  Family: bernoulli 
##   Links: mu = logit 
## Formula: SUI_CON ~ VIOLENCEcwc * ES + VIOLENCEcwc * ES_smean + STRESS + HEALTH_cont + SAD + GRADE_cont + SEX + RESIDENTIAL + (1 + VIOLENCEcwc | SCHOOL) 
##    Data: bigdat2s (Number of observations: 179619) 
## Samples: 4 chains, each with iter = 3000; warmup = 1000; thin = 1;
##          total post-warmup samples = 8000
## 
## Group-Level Effects: 
## ~SCHOOL (Number of levels: 2399) 
##                            Estimate Est.Error l-95% CI u-95% CI Rhat Bulk_ESS Tail_ESS
## sd(Intercept)                  0.23      0.01     0.20     0.26 1.00     2550     3586
## sd(VIOLENCEcwc)                0.62      0.10     0.42     0.80 1.00     1266     1648
## cor(Intercept,VIOLENCEcwc)     0.09      0.15    -0.18     0.39 1.00     1226     1867
## 
## Population-Level Effects: 
##                             Estimate Est.Error l-95% CI u-95% CI Rhat Bulk_ESS Tail_ESS
## Intercept                      -3.69      0.02    -3.74    -3.64 1.00     5632     6100
## VIOLENCEcwc                     0.91      0.07     0.76     1.05 1.00     3729     5140
## ESlow                           0.46      0.05     0.37     0.54 1.00     9361     6089
## ESmidMlow                       0.32      0.02     0.27     0.37 1.00     8511     6180
## ESmidMhigh                     -0.00      0.02    -0.04     0.04 1.00     8354     5991
## EShigh                          0.02      0.03    -0.04     0.08 1.00     8076     6162
## ES_smean                       -0.00      0.01    -0.03     0.02 1.00     5821     6327
## STRESSnone                     -0.19      0.07    -0.33    -0.04 1.00     9103     6149
## STRESSlow                      -0.64      0.05    -0.73    -0.55 1.00     7711     5868
## STRESShigh                      0.90      0.02     0.86     0.95 1.00     5735     5716
## STRESSveryhigh                  1.62      0.02     1.57     1.67 1.00     5308     5961
## HEALTH_cont                    -0.24      0.01    -0.26    -0.22 1.00     9018     6032
## SAD1                            1.91      0.02     1.88     1.95 1.00     7647     6077
## GRADE_cont                     -0.10      0.01    -0.11    -0.09 1.00     7349     6427
## SEX                             0.24      0.02     0.20     0.28 1.00     7531     6029
## RESIDENTIALrelative             0.50      0.08     0.35     0.66 1.00    10217     6547
## RESIDENTIALdormDstudio          0.10      0.04     0.02     0.17 1.00     9256     6025
## RESIDENTIALnursuryDorphange     0.25      0.14    -0.02     0.53 1.00     9105     6053
## VIOLENCEcwc:ESlow               0.37      0.16     0.05     0.69 1.00     5997     6107
## VIOLENCEcwc:ESmidMlow           0.07      0.13    -0.19     0.33 1.00     5521     5577
## VIOLENCEcwc:ESmidMhigh          0.19      0.11    -0.03     0.41 1.00     4916     5331
## VIOLENCEcwc:EShigh              0.45      0.12     0.21     0.68 1.00     5486     5797
## VIOLENCEcwc:ES_smean           -0.09      0.05    -0.20     0.01 1.00     7293     6380
## 
## Samples were drawn using sampling(NUTS). For each parameter, Bulk_ESS
## and Tail_ESS are effective sample size measures, and Rhat is the potential
## scale reduction factor on split chains (at convergence, Rhat = 1).
```

Plot the results (Fig 5). I will just plot the predicted probabilities analogous to Fig 2.

I am making predictions for a hypothetical school whose proportion of the crisis dummy variable involved in the interaction is equal to the proportion in the overall population. As those dummies were cluster-mean centered in the MLM regressions, a value of ‘-overall mean’ in a cluster-mean centered dummy indicates a 0 in the original dummy (e.g. absence of recent despair), and a value of ‘1-overall mean’ indicates a 1 in the original dummy (e.g. presence of recent despair). Random effects were set to zero for plotting.

```
#sadxstr
meansad <- mean(bigdat2s$SAD_numeric)

newdat.s1 <- data.frame(
  "SADcwc"= c( rep(  (-meansad),5),  rep((1-meansad),5)   ),
  "STRESS"= c(rep(  c("very high","high", "some", "low", "none"),2) ),
  "STRESS_smean"= c(rep(0,10) ),
  "ES"= c(rep("mid",10) ),
  "HEALTH_cont"= c(rep(0,10) ), 
  "VIOLENCE" = c( rep("0",10) ),
  "SEX" = c( rep(0,10) ),
  "GRADE_cont" = c(rep(0, 10)),
  "RESIDENTIAL" = c(rep("family", 10))
)

pred <- fitted(brm_sadxstr1, newdata = newdat.s1, probs = c(0.05, 0.95), re_formula = NA)

df <- data.frame( "DESPAIR" = c(rep("0",5), rep("1",5)),
                  "STRESS" = c( "very high","high","some","low","none", "very high","high","some","low","none")
)

dats1 <- cbind(df, pred)
dats1$STRESS <- factor( dats1$STRESS, levels = c("very high","high","some","low","none")  )

graphs1 <- dats1 %>% 
  ggplot(aes(x = STRESS, 
             y = Estimate, 
             color = DESPAIR,
             ymin=Q5,
             ymax=Q95)) +
  geom_point(size=2.5) +
  geom_errorbar(width = 0.55, size=1) +
  ylim(0, 0.5) +
  labs(x = "Stress",
       color  = "Despair/Grief",
       y = "Probability of Suicidal Ideation") +
  theme_minimal() +
  ggtitle("Model 2 MLM") +
  theme(plot.title = element_text(colour="black", size=11.5),
        legend.text = element_text(colour="black",size=10), legend.title = element_text(face="plain",colour="black", size=10),legend.position="bottom",
        axis.title.x = element_text(face="plain",colour="black", size=11.5), axis.title.y = element_text(face="plain",colour="black", size=10),
        axis.text.x=element_text(size=10, color = "black", angle=45), axis.text.y=element_text(size=10, color = "black")) +
  scale_color_manual(labels = c("0"= "No", "1"=  "Yes"), values=c("blue","red"))


#violxstr
meanviol <- mean(bigdat2s$VIOLENCE_numeric)

newdat.v1 <- data.frame(
  "VIOLENCEcwc"= c( rep(  (-meanviol),5),  rep((1-meanviol),5)   ),
  "STRESS"= c(rep(  c("very high","high", "some", "low", "none"),2) ),
  "STRESS_smean"= c(rep(0,10) ),
  "ES"= c(rep("mid",10) ),
  "HEALTH_cont"= c(rep(0,10) ), 
  "SAD" = c( rep("0",10) ),
  "SEX" = c( rep(0,10) ),
  "GRADE_cont" = c(rep(0, 10)),
  "RESIDENTIAL" = c(rep("family", 10))
)

pred <- fitted(brm_violxstr1, newdata = newdat.v1, probs = c(0.05, 0.95), re_formula = NA)

df <- data.frame( "VIOLENCE" = c(rep("0",5), rep("1",5)),
                  "STRESS" = c( "very high","high","some","low","none", "very high","high","some","low","none")
)

datv1 <- cbind(df, pred)
datv1$STRESS <- factor( datv1$STRESS, levels = c("very high","high","some","low","none")  )

graphv1 <- datv1 %>% 
  ggplot(aes(x = STRESS, 
             y = Estimate, 
             color = VIOLENCE,
             ymin=Q5,
             ymax=Q95)) +
  geom_point(size=2.5) +
  geom_errorbar(width = 0.55, size=1) +
  ylim(0, 0.25) +
  labs(x = "Stress",
       color  = "Violence",
       y = "Probability of Suicidal Ideation") +
  theme_minimal() +
  ggtitle("Model 3 MLM") +
  theme(plot.title = element_text(colour="black", size=11.5),
        legend.text = element_text(colour="black",size=10), legend.title = element_text(face="plain",colour="black", size=10),legend.position="bottom",
        axis.title.x = element_text(face="plain",colour="black", size=11.5), axis.title.y = element_text(face="plain",colour="black", size=10),
        axis.text.x=element_text(size=10, color = "black", angle=45), axis.text.y=element_text(size=10, color = "black")) +
  scale_color_manual(labels = c("0"= "No", "1"=  "Yes"), values=c("blue","red"))


#sadxes
newdat.s3 <- data.frame(
  "SADcwc"= c( rep(  (-meansad),5),  rep((1-meansad),5)   ),
  "ES"= c( rep(  c("high","mid-high","mid","mid-low","low"),2) ),
  "ES_smean" = c(rep(0,10) ),
  "STRESS"= c(rep("some",10) ), 
  "HEALTH_cont"= c(rep(0,10) ),
  "VIOLENCE" = c( rep("0",10) ),
  "SEX" = c( rep(0,10) ),
  "GRADE_cont" = c(rep(0, 10)),
  "RESIDENTIAL" = c(rep("family", 10))
)

pred <- fitted(brm_sadxes1, newdata = newdat.s3, probs = c(0.05, 0.95), re_formula = NA)

df <- data.frame( "DESPAIR" = c(rep("0",5), rep("1",5)),
                  "ES" = c( "high","mid-high","mid","mid-low","low","high","mid-high","mid","mid-low","low")
)
dats3 <- cbind(df, pred)
dats3$ES <- factor( dats3$ES, levels = c("low","mid-low","mid","mid-high","high")  )

graphs3 <- dats3 %>% 
  ggplot(aes(x = ES, 
             y = Estimate, 
             color = DESPAIR,
             ymin=Q5,
             ymax=Q95)) +
  geom_point(size=2.5) +
  geom_errorbar(width = 0.55, size=1) +
  ylim(0, 0.25) +
  labs(x = "Economic Status",
       color  = "Despair/Grief",
       y = "Probability of Suicidal Ideation") +
  theme_minimal() +
  ggtitle("Model 4 MLM") +
  theme(plot.title = element_text(colour="black", size=11.5),
        legend.text = element_text(colour="black",size=10), legend.title = element_text(face="plain",colour="black", size=10),legend.position="bottom",
        axis.title.x = element_text(face="plain",colour="black", size=11.5), axis.title.y = element_text(face="plain",colour="black", size=10),
        axis.text.x=element_text(size=10, color = "black", angle=45), axis.text.y=element_text(size=10, color = "black")) +
  scale_color_manual(labels = c("0"= "No", "1"=  "Yes"), values=c("#00AFBB","#FC4E07"))


#compute the p-value of the difference between high and mid when Despair = Yes. That amounts to checking if the sum of the main and interaction effect of "high" economic status is different from zero.
sum <- fixef(brm_sadxes1)[22]+fixef(brm_sadxes1)[6]
sd <- sqrt( vcov(brm_sadxes1)[22,22] + vcov(brm_sadxes1)[6,6] + 2*vcov(brm_sadxes1)[22,6])
z <- sum/sd 
(p <- 2*pnorm(-abs(z)))
```

```
## [1] 0.001932634
```

```
#violxes
newdat.v3 <- data.frame(
  "VIOLENCEcwc"= c( rep(  (-meanviol),5),  rep((1-meanviol),5)   ),
  "ES"= c( rep(  c("high","mid-high","mid","mid-low","low"),2) ),
  "ES_smean" = c(rep(0,10) ),
  "STRESS"= c(rep("some",10) ), 
  "HEALTH_cont"= c(rep(0,10) ),
  "SAD" = c( rep("0",10) ),
  "SEX" = c( rep(0,10) ),
  "GRADE_cont" = c(rep(0, 10)),
  "RESIDENTIAL" = c(rep("family", 10))
)

pred <- fitted(brm_violxes1, newdata = newdat.v3, probs = c(0.05, 0.95), re_formula = NA)

df <- data.frame( "VIOLENCE" = c(rep("0",5), rep("1",5)),
                  "ES" = c( "high","mid-high","mid","mid-low","low","high","mid-high","mid","mid-low","low")
)

datv3 <- cbind(df, pred)
datv3$ES <- factor( datv3$ES, levels = c("low","mid-low","mid","mid-high","high")  )

graphv3 <- datv3 %>% 
  ggplot(aes(x = ES, 
             y = Estimate, 
             color = VIOLENCE,
             ymin=Q5,
             ymax=Q95)) +
  geom_point(size=2.5) +
  geom_errorbar(width = 0.55, size=1) +
  ylim(0, 0.20) +
  labs(x = "Economic Status",
       color  = "Violence",
       y = "Probability of Suicidal Ideation") +
  theme_minimal() +
  ggtitle("Model 5 MLM") +
  theme(plot.title = element_text(colour="black", size=11.5),
        legend.text = element_text(colour="black",size=10), legend.title = element_text(face="plain",colour="black", size=10),legend.position="bottom",
        axis.title.x = element_text(face="plain",colour="black", size=11.5), axis.title.y = element_text(face="plain",colour="black", size=10),
        axis.text.x=element_text(size=10, color = "black", angle=45), axis.text.y=element_text(size=10, color = "black")) +
  scale_color_manual(labels = c("0"= "No", "1"=  "Yes"), values=c("#00AFBB","#FC4E07"))


g1 <- ggarrange(graphs1, graphv1,
                labels = c("a", "b"),
                ncol = 2, nrow=1, font.label = list(size = 11.5), legend="top")

g2 <- ggarrange(graphs3, graphv3,
                labels = c("c", "d"),
                ncol = 2, nrow=1, font.label = list(size = 11.5), legend="top")
```

```
(g3 <-ggarrange(g1, g2, ncol=2, nrow=1))
```

Let’s also try plotting the interaction between recent despair and school mean economic status as briefly discussed in the main text.

```
#sadxes (groupmean)
newdat.s <- data.frame(
  "SADcwc"= c( rep(  (-meansad),100),  rep((1-meansad),100)   ),
  "ES"= c(rep("mid",200)),
  "ES_smean" = c( rep(seq(from=min(bigdat2s$ES_smean), to=max(bigdat2s$ES_smean), length.out = 100),2 ) ),
  "STRESS"= c(rep("some",200) ), 
  "HEALTH_cont"= c(rep(0,200) ),
  "VIOLENCE" = c( rep("0",200) ),
  "SEX" = c( rep(0,200) ),
  "GRADE_cont" = c(rep(0, 200)),
  "RESIDENTIAL" = c(rep("family", 200))
)

pred <- fitted(brm_sadxes1, newdata = newdat.s, probs = c(0.05, 0.95), re_formula = NA, scale="response") #on a logit scale, we surely see the slight difference in slope. But on a probability scale, the two lines look nearly parallel.

df <- data.frame( "DESPAIR" = c(rep("0",100), rep("1",100)),
                  "School Mean ES" = c( rep(seq(from=min(bigdat2s$ES_smean), to=max(bigdat2s$ES_smean), length.out = 100),2 ) )
)
dats <- cbind(df, pred)

graphs <- dats %>% 
  ggplot(aes(x = School.Mean.ES, 
             y = Estimate, 
             color = DESPAIR)) +
  geom_line(size=0.8) +
  labs(x = "School Mean Economic Status (Z-score)",
       color  = "Despair/Grief",
       y = "Probability of Suicidal Ideation") +
  theme_minimal() +
  ggtitle("Model 4 MLM") +
  theme(plot.title = element_text(colour="black", size=11.5),
        legend.text = element_text(colour="black",size=10), legend.title = element_text(face="plain",colour="black", size=10),legend.position="top",
        axis.title.x = element_text(face="plain",colour="black", size=11.5),axis.title.y = element_text(face="plain",colour="black", size=10),
        axis.text.x=element_text(size=10, color = "black"), axis.text.y=element_text(size=10, color = "black")) +
  scale_color_manual(labels = c("0"= "No", "1"=  "Yes"), values=c("#00AFBB","#FC4E07"))
```

```
graphs
```
