# Supplementary material for: Do more stress and lower family economic status increase vulnerability to suicidal ideation? Evidence of a U-shaped relationship in a large cross-sectional sample of South Korean adolescents
Source: PLoS One. 2021 Apr 26;16(4):e0250794. doi: 10.1371/journal.pone.0250794 (PMC8075251; doi:10.1371/journal.pone.0250794)
Supplement: Attachment — Submitted filename: Response to Reviewers.docx [file pone.0250794.s004.docx]

**Response to Reviewers**

PONE-D-20-36724R1

April 6, 2021

Dear Dr. Vincenzo De Luca,

Editor of PLOS ONE,

I have received minor but apt criticisms from the anonymous reviewer and made certain changes to the manuscript. Listed below are direct citations of the reviewer’s comments followed by my responses.

1) Line 206: Please describe how you handled the missing values for those variables that had them? What were the statistical decisions made for those variables with missing values?

Author’s Response: Thank you for pointing this out. In response to this comment, I explained that there are almost no missing values in the entire dataset because students were required to answer all questions in the online survey, and that the rare cases of missing responses come from a handful of confidential questions in the 2019 survey. I also clearly declared that missing data posed no concerns for variable choice given the excellent availability of virtually all relevant variables.

2) Lines 476/480: See if you can rephrase the "harder" and "softer" referenced in this paragraph of the discussion section. It is too colloquial and contains certain judgements/connotations that did not fit the rigour of a scientific discussion. I understood what you meant by it, but see if you can reword it and explain it in a different way for the lay audience.

Author’s Response: I deleted these words and used expressions that I believe are more suitable for academic communication.

I would like to thank Dr. De Luca and the anonymous reviewer again for helping me improve and (potentially) publish my paper at PLOS ONE.

Yours sincerely,

Tay Jeong
